# Brain tumour differentiation: rapid stratified serum diagnostics via attenuated total reflection Fourier-transform infrared spectroscopy

**DOI:** 10.1007/s11060-016-2060-x

**Published:** 2016-02-13

**Authors:** James R. Hands, Graeme Clemens, Ryan Stables, Katherine Ashton, Andrew Brodbelt, Charles Davis, Timothy P. Dawson, Michael D. Jenkinson, Robert W. Lea, Carol Walker, Matthew J. Baker

**Affiliations:** WestCHEM, Department of Pure and Applied Chemistry, Technology and Innovation Centre, University of Strathclyde, 99 George Street, Glasgow, G11RD UK; Centre for Materials Science, Division of Chemistry, University of Central Lancashire, Preston, PR12HE UK; Digital Media Technology Laboratory, Millennium Point, City Centre Campus Birmingham City University, West Midlands, B47XG UK; Neuropathology, Lancashire Teaching Hospitals NHS Trust, Royal Preston Hospital, Sharoe Green Lane North, Preston, PR29HT UK; The Walton Centre for Neurology and Neurosurgery, The Walton Centre NHS Trust, Lower Lane, Liverpool, L97LJ UK; School of Pharmacy and Biomedical Sciences, Maudland Building, University of Central Lancashire, Preston, PR12HE UK

**Keywords:** ATR-FTIR, Serum, Diagnostics, Cancer, Glioma, Spectroscopy, Rapid

## Abstract

The ability to diagnose cancer rapidly with high sensitivity and specificity is essential to exploit advances in new treatments to lead significant reductions in mortality and morbidity. Current cancer diagnostic tests observing tissue architecture and specific protein expression for specific cancers suffer from inter-observer variability, poor detection rates and occur when the patient is symptomatic. A new method for the detection of cancer using 1 μl of human serum, attenuated total reflection—Fourier transform infrared spectroscopy and pattern recognition algorithms is reported using a 433 patient dataset (3897 spectra). To the best of our knowledge, we present the largest study on serum mid-infrared spectroscopy for cancer research. We achieve optimum sensitivities and specificities using a Radial Basis Function Support Vector Machine of between 80.0 and 100 % for all strata and identify the major spectral features, hence biochemical components, responsible for the discrimination within each stratum. We assess feature fed-SVM analysis for our cancer versus non-cancer model and achieve 91.5 and 83.0 % sensitivity and specificity respectively. We demonstrate the use of infrared light to provide a spectral signature from human serum to detect, for the first time, cancer versus non-cancer, metastatic cancer versus organ confined, brain cancer severity and the organ of origin of metastatic disease from the same sample enabling stratified diagnostics depending upon the clinical question asked.

**Electronic supplementary material:**

The online version of this article (doi:10.1007/s11060-016-2060-x) contains supplementary material, which is available to authorized users.

## Introduction

Attenuated total reflection—Fourier transform infrared spectroscopy is rapid, cost-effective, simple to operate and can be handheld. Biomolecules exhibit responses to different wavelengths of light, the resulting spectrum can be thought of as the sample ‘fingerprint’, spectroscopic analysis allows for objective classification on a molecular level [1]. ATR-FTIR is an excellent vibrational spectroscopic technique for the analysis of biofluids (e.g. serum) due to its rapidity and ease of translation to the clinical environment, i.e. ATR-FTIR requires no sample preparation when analysing serum [2]. During ATR-FTIR the infrared light is directed through an internal reflection element (IRE) with a high refractive index (e.g. diamond/germanium) enabling an evanescent-wave to extend beyond the IRE surface penetrating the sample, which must be in intimate contact with the IRE surface [2]. A rapid spectroscopic serum-screening regime would significantly reduce current diagnosis times and greatly increase the chance of successful treatments [3–4]. Blood serum is a primary carrier of small molecules in the body; it holds all secreted molecules from different tissues in response to different physiological needs, dysfunctions and pathological states [5].

Currently, in the UK, 38 % of people living with a brain tumour visited their GP more than five times before being diagnosed [6]. In addition, 23 % of newly diagnosed cancer patients came from emergency presentations, with 1 year survival rates much lower than those diagnosed via other routes [6].Current diagnosis relies upon time consuming and subjective histopathological examination. Diagnostic error occurs in up to 50 % of cases, which can result in additional testing, diagnostic delays and incorrect diagnoses [7]. Prior to diagnosis the patient will have to be symptomatic in order to be referred. Metastatic brain diseases are the most common form of intracranial neoplasm in adults and are predicted to develop in 20–40 % of cancer patients [8]. Identifying the primary site of origin increased the therapeutic success, however, in approximately 15 % of metastatic cancer cases the location of the primary is unknown [8]. Blood is the most ubiquitous fluid used for diagnosis. Most current blood tests detect single biomarkers that are of limited suitability for screening [9], as cancer is a heterogeneous disease a set of markers would provide significantly more information that any one marker.

Previous spectroscopic research has provided evidence of the benefits of applying spectroscopy to clinical problems [8, 10], and recently to the spectroscopic diagnosis of diseases via biofluid analysis [11–12]. We have shown the potential of ATR-FTIR spectroscopy for the rapid diagnosis of brain tumour severity using a 1 μl volume of patient serum and within 10 min enabling diagnosis of high grade glioma, low grade glioma and non-cancer with severities and specificities on average of 93.75 and 96.53 % respectively [11–12]. Ollesch et al. have developed a robotic spotting system in combination with vacuum drying for the application of blood-derived substances which would offer the ability of rapid screening [13].

A number of studies assess the role of spectroscopy for the diagnosis of disease. Owens et al. successfully discriminated between patients with ovarian cancer and non-cancer using blood serum and plasma with Raman and ATR-FTIR spectroscopy [14]. Gajjar et al. has shown the ability of ATR-FTIR to differentiate between patients diagnosed with either ovarian or endometrial cancer from non-cancer controls using blood serum samples. Classification results were as high as 96.7 % for ovarian cancer and 81.7 % for endometrial cancer [15]. Backhaus et al. distinguished between breast cancer serum and non-cancer controls achieving a sensitivity and specificity of 98 and 95 % respectively [16].

This study reports, for the first time, the ability to provide stratified multiple diagnoses from human serum to greatly enhance the capability and information obtained for a simple, effective, reproducible and repeatable technique. We report the application of ATR-FTIR spectroscopy for stratified serum spectroscopic diagnostics capable of diagnosing at different levels; from general cancer versus non-cancer, metastatic cancer versus primary brain cancer, glioma versus meningioma, the severity of the tumour (high-grade glioma vs. low-grade glioma) and the organ of origin of brain metastases using only 1 µl of a patient serum sample and within 10 min of serum application. This will provide a rapid diagnostic process capable of deployment in situ from primary to tertiary care systems depending upon the information required by different clinical settings.

## Materials and methods

### Serum samples

Blood samples were collected from 433 patients over the range of cancer groups analysed. Table 1 provides demographic information based on cancer group. The average age is 57.77 and 44.77 years for the cancer and non-cancer patient sample sets, respectively. Full sample data can be found in supplementary information Table S2. The research described in this paper was performed with full ethical approval (Walton Research Bank BTNW/WRTB 13_01/BTNW Application #1108). All blood samples were collected pre-operatively. The serum tubes were left to clot at room temperature for a minimum of 30 min and maximum of 2 h from blood draw to centrifugation. Separation of the clot was accomplished by centrifugation at 1,200 g for 10 min and 500 μl aliquots of serum dispensed. All serum samples were snap frozen using liquid nitrogen and stored at −80 °C. S1 shows a flow diagram of the analysis and pre-processing steps, including which patient samples are in each classification. Non-cancer (control) serum samples were collected from individuals who presented no symptoms of cancer at a Royal Preston Hospital (UK) blood donation event, as well as those presenting to the clinic for elective surgery.

**Table 1:** Total subject number of tumour grade, age range, mean age and gender of patient samples

Tumour grade	Number of subjects	Age range/mean age	Gender
Non-cancer	122	16–89/44.77 years	64 Male, 58 female
All cancer	311	19–82/57.77 years	133 Male, 178 female
Glioma	87	19–81/49.90 years	52 Male, 35 female
Low-grade glioma	23	19–60/38.35 years	11 Male, 12 female
High-grade glioma	64	25–81/61.44 years	41 Male, 23 female
Meningioma	47	24–78/55.98 years	13 Male, 34 female
Metastasis	177	25–82/59.45 years	68 Male, 109 female
Lung metastasis	84	25–82/59.32 years	36 Male, 48 female
Breast metastasis	36	27–76/50.92 years	0 Male, 36 female
Melanoma Metastasis	25	25–80/56.00 years	14 Male, 11 female

We previously investigated the reproducibility of the serum spectrum and the length of time required for a reproducible spectrum to be obtained from a 1 μl volume of whole serum. At room temperature, 1 μl of serum has been found to dry after 8 min through repeat drying experiments. The reproducibility of serum spectral data using ATR-FTIR is high and exhibits minimal variance, especially after pre-processing, for 150 spectra collected from 50 different human pool serum spots (3 spectral repeats per spot). We found that the largest variance of the ATR-FTIR spectrum was at 1637.27 cm^−1^ with a standard deviation (STD) of 0.0050 and the smallest variance at 3735.33 cm^−1^ with a STD of 0.0038 from 150 collected spectra. After noise reduction (30 principal components) and vector normalization these STD values were reduced to 0.0043 and 0.00123 respectively [12].

### Instrumentation

All spectra were collected using an Agilent Cary-600 Series FTIR spectrometer with a PIKE Technologies MIRacle™ single-reflection ATR configured with a diamond (Di) crystal plate. 1 μl volumes of human serum were pipetted onto the ATR-FTIR crystal using an Eppendorf Research-Plus 0.5–10.0 ul pipette. After spectral collection from each 1 μl dried serum spot, Virkon disinfectant (fisher-scientific) and 99.5 % ethanol (thermo-scientific) were used consecutively to remove the serum film from the crystal.

### ATR-FTIR diagnostic model

All whole serum samples were thawed prior to spectral collection at room temperature. Spectra were collected in a random order within the serum sample sets. For each sample, a 1 μl serum spot was pipetted onto the ATR-FTIR crystal and allowed to dry for 8 min, at which time three spectra were collected. Prior to spectral collection, a background absorption spectrum was collected (for atmospheric correction) before the 1 μl of serum was pipetted onto the ATR-FTIR crystal. A single background was collected per sample replicate. Spectra were acquired in the range of 4000–600 cm^−1^, at a resolution of 4 cm^−1^ and averaged over 32 co-added scans. In total, 3897 ATR-FTIR spectra were collected from all serum samples.

### Data handling and analysis

Initially agilent’s resolutions-pro FTIR software was used for data handling after which the spectra were imported for further analysis and processing into Matlab™ using in–house written and open source protocols.

For all spectra acquired, the fingerprint region (1800–1000 cm^−1^) was selected for multivariate analysis. A principal component based noise reduction, using the first 50 principal components of the data was performed on the spectra to improve the signal-to-noise ratio. Following noise reduction, all spectra were vector normalised. Using LIBSVM and in-house written protocols [www.csie.ntu.edu.tw/~cjlin/libsvm] in MATLAB™, an *n*-fold cross validation was performed (*n* = 5) on the training data to determine the optimum values for the cost and gamma functions. Supplementary information S3 shows the optimum cost and gamma functions for each stratum (e.g. cancer vs. non-cancer). The optimum cost and gamma values were used to train the support vector machine (SVM) in a one-versus-rest mode using a randomly selected training set consisting of 2/3 of the patient associated spectral data. The remainder of the data (1/3) was used to create the test set which was then projected into the model, and confusion matrices were calculated giving an overall SVM classification based on the true and predicted data class labels. For each stratum 525 combinations of 2/3 training and 1/3 test were performed based upon patient membership, thus, all spectra from one patient was either in the train set or the test set. Sensitivities and specificities were calculated for each combination in order to understand the effect of patient membership in test and training sets based upon sensitivity and specificity.

### Feature extraction

The main function of feature extraction is to elucidate and rank the relevant discriminatory spectral information from recorded ATR-FTIR data. Per stratum, all pre-processed spectral data were variably ranked (30 %) with information gain. Variable ranking highlights the wavenumber variables that are most salient between the spectral classes. In the case of the cancer versus non-cancer stratum, 130 wavenumber variables associated to six spectral regions were selected (Table 4). Following variable ranking, the ranked wavenumber regions were user selected on a 2D plot of the mean spectrum, upon which feature extraction (FE) was performed. FE was performed whereby spectral descriptors such as RMS energy, peak kurtosis, peak skew, peak centroid, peak frequency and peak amplitude can be extracted from each user selected spectral band, thus the relevant spectral band shapes involved in the discrimination between classes are able to be captured. The feature information is ranked and scored in descending order to describe how each feature of the model explains the difference between the groups of recorded spectral data. The most discriminatory features highlighted during feature extraction were then used for a feature based SVM (FE-SVM). Using LIBSVM and in house written protocols [www.csie.ntu.edu.tw/~cjlin/libsvm] in MATLAB™, an *n*-fold cross validation was performed (*n* = 5) on the cancer versus non-cancer spectral training data to determine the optimum values for the cost and gamma functions. FE-SVM was performed using all 130 spectral features followed by the top 30 and top 2 features for the cancer versus non-cancer data set.

### Sensitivity and Specificity

Sensitivity and specificity were calculated using Eqs. 1 and 2 respectively:

1 $$ Sensitivity = \frac{True\;Positives}{True\;Positives + False\;Negatives} $$

2 $$ Specificity = \frac{True\;Negatives}{True\;Negatives + False\;Positives} $$

where,True Positives is the a patient with the target disease has five or more spectra out of the nine spectra collected from three different serum spots (three spectra per spot) correctly identified.

True Negatives is the a patient without the target disease who has five or more spectra out of the nine spectra collected from three different serum spots (three spectra per spot) correctly identified.

False Positives is the a patient without the target disease who has five or more spectra out of the nine spectra collected from three different serum spots (three spectra per spot) that have been incorrectly identified as the target disease.

False Negatives is the a patient with the target disease who has five or more spectra out of the nine spectra collected from three different serum spots (three spectra per spot) that have been incorrectly classified as not the target disease.

### Kappa values

Kappa values were calculated using Eq. 3:

3 $$ K = \frac{{(p_{o} - p_{e} )}}{{(1 - p_{e} )}} $$

where, K is the Kappa Value, P_o_ is the observed agreement, P_e_ is the expected agreement (chance agreement), p_o_ and p_e_ were calculated using Eqs. 4 and 5 respectively

4 $$ \frac{(TP + TN)}{(TP + TN + FP + FN)} $$

5 $$ \begin{aligned} \left[ {\left( {{\raise0.7ex\hbox{${TP + FP}$} \!\mathord{\left/ {\vphantom {{TP + FP} {SUM\,ALL}}}\right.\kern-0pt} \!\lower0.7ex\hbox{${SUM\,ALL}$}}} \right) \times \left( {{\raise0.7ex\hbox{${TP + FN}$} \!\mathord{\left/ {\vphantom {{TP + FN} {SUM\,ALL}}}\right.\kern-0pt} \!\lower0.7ex\hbox{${SUM\,ALL}$}}} \right)} \right] \hfill \\ \quad + \left[ {\left( {{\raise0.7ex\hbox{${FN + TN}$} \!\mathord{\left/ {\vphantom {{FN + TN} {SUM\,ALL}}}\right.\kern-0pt} \!\lower0.7ex\hbox{${SUM\,ALL}$}}} \right) \times \left( {{\raise0.7ex\hbox{${FP + TN}$} \!\mathord{\left/ {\vphantom {{FP + TN} {SUM\,ALL}}}\right.\kern-0pt} \!\lower0.7ex\hbox{${SUM\,ALL}$}}} \right)} \right] \hfill \\ \end{aligned} $$

where, TP is the true positives, TN is the true negatives, FP is the false positives, FN is the false negatives, SUM ALL is the TP + TN + FP + FN.

Using a patient based spectral diagnosis (correct classification of at least five out of nine spectra from three different patient serum spots) when compared to clinical diagnosis of that patient following a multidisciplinary team (MDT) meeting.

## Results and discussion

### Rapid stratified serum spectroscopic diagnostics

ATR-FTIR spectra from 433 patients (3897 spectra) were analysed to investigate sensitivities and specificities possible on a patient level. 525 iterations with different training and test spectral datasets (split 1/3 test and 2/3 training on a patient basis) were used to analyze the power of the RBF-SVM analysis. Supplementary information S4 displays a histogram of the range of sensitivities and specificities achieved for the cancer versus non-cancer stratum (histograms for all other strata are displayed in supplementary information S5). The sensitivity and specificity range for cancer versus non-cancer is 81–97 % and 51–95 % respectively with sensitivity and specificity ranges of 46–80 % and 60–93 % respectively for metastatic cancer versus brain cancer, 48–100 % and 31–100 % respectively for glioma versus meningioma, 50–100 % and 2–100 % respectively for high-grade glioma versus low-grade glioma and 28–95 % and 68–98 % for the metastatic origin stratum. Table 2 shows the mean, mode and optimum sensitivities and specificities for each stratum. The optimum sensitivity and specificity is the sensitivity and specificity that best describes the sample set based upon disease grouping.

**Table 2:** Mean, mode and optimum sensitivities and specificities obtained for each stratum

Model	Optimum sensitivity (%)	Optimum specificity (%)	Mean sensitivity (%)	Mean specificity (%)	Mode sensitivity (%)	Mode specificity (%)
Cancer versus non-cancer	97.1	95.1	89.8	77.5	89.4	78.0
Metastatic cancer versus brain cancer	80.0	93.2	79.7	64.0	64.4	80.0
Glioma versus meningioma	100.0	100.0	81.1	66.7	82.1	75.0
High grade glioma (HGG) versus low grade glioma (LGG)	100.0	100.0	80.9	48.5	85.0	50.0

Metastatic model	Optimum sensitivity (%)	Optimum specificity (%)	Mean sensitivity (%)	Mean specificity (%)	Mode sensitivity (%)	Mode specificity (%)
Metastatic lung cancer	95.4	95.9	79.0	85.7	81.4	84.9
Metastatic skin cancer	84.4	94.4	63.9	82.0	64.4	80.3
Metastatic breast cancer	78.6	98.9	51.4	90.1	50.0	90.9
Metastatic model mean	86.3	98.3	64.8	86.0	65.3	85.4

The optimum, mode and mean sensitivities and specificities observed for all strata range from 51.4 to 100 % respectively, with the optimum sensitivities and specificities achieving 86.3–100 %. The cancer versus non-cancer stratum achieved a mean sensitivity and specificity of 89.8 and 77.5 % respectively, metastatic cancer versus brain cancer of 79.7 and 64.0 % respectively, glioma versus meningioma of 66.7 and 82.1 % respectively, high grade glioma versus low grade glioma of 80.9 and 48.5 % respectively and the origin of metastasis of 64.8 and 86.9 % respectively.

These results show the power of ATR-FTIR spectroscopy to diagnose disease states based upon a stratified approach; however variance still exists in the spectral datasets due to the selection of patient populations in the test and training set. For each stratum, sensitivity and specificity variance exists between classification model iterations. This shows that certain patient partitions provide better classification for the remaining test patient data set. A reason for this is redundant data maximizing the spectral variance within a group within the data variables of the spectral fingerprint region i.e. patient data containing higher intra-group spectral variance partitioned together to form the training set would produce poorer classification models.

### Feature extraction for stratified serum spectroscopic diagnostics

To maximize classification accuracy the most salient features of a spectrum can be extracted and ranked based on their similarity to a target set, thus assigning scores on the feature’s ability to discriminate between classes, maximizing inter-group differences [17].The spectral features used are the peak centroid (measure of the peak’s central point), peak skew (measure of asymmetry in the peak’s shape), peak kurtosis (a measure of the shape of a peak relating peaked vs. flat-topped), peak amplitude and root-mean-squared (RMS) energy. These features were extracted from pre-defined sub-bands of each spectrum and the corresponding inter-band ratios between features were then ranked, using the information gain metric, based upon the resulting score.

Following feature extraction and variable ranking the most discriminatory characteristics of the spectrum (from 1800 to 900 cm^−1^) were extracted (Table 3 displays the most discriminatory regions with proposed biomolecular assignments) highlighting spectral components relating to proteins, lipids, carbohydrates and nuclear material.

**Table 3:** Discriminatory spectral regions with biomolecular assignments

Wavenumber region (cm^−1^)	Assignments
1008–1230	C–O stretch, deoxyribose/ribose, DNA, RNA (PO_2_^−^), C–C stretch, C–H bend
1315–1384	CH_3_/CH_2_ bending
1380–1465	CH_3_ lipids/proteins and COO^−^ of amino acids
1460–1590	Amide II of proteins (α—helix structures*, β*—pleated sheet structures, turns, random coils), δ N–H (60 %), ν C–N (40 %)
1600–1706	Amide I of proteins (α—helix structures*, β*—pleated sheet structures, turns, random coils), ν C=O (76 %), ν C–N (14 %), CNN (10 %)
1700–1799	δ C=O of lipids

Interestingly the features observed for the 2-class strata, enabling classification of cancer versus non-cancer, metastatic versus brain cancer, glioma versus meningioma and high-grade glioma versus low-grade glioma (top 10 features for each 2-class stratum are displayed in supplementary information S6) which focus on the detection and diagnosis of primary brain cancer are originating from the Amide I (vibrations originating from α–helix structures, β-pleated sheets, turns and random coil (νC = O (80 %), νC–N (10 %), CNN (10 %)) [18] and Amide II—vibrations originating from α–helix structures, β-pleated sheets, turns and random coil [δ N–H (60 %), ν C–N (40 %)], C–O stretch of lipids/proteins, CH_2_ of lipids/proteins and contributions from nuclear materials (DNA/RNA via PO_2_^−^ stretches) spectral regions [18–27]. These spectral regions have been described previously in research discriminating between brain cancer states using tissue spectroscopy [22, 25]. The former highlighted the Amide I (1655 cm^−1^), Amide II (1547 and 1582 cm^−1^), carbohydrate (1173 cm^−1^), glycogen (1014 cm^−1^) and phosphate regions as describing the majority of difference between infrared spectra of tissue origination from non-cancerous patients and tumour subtypes.

The features observed for the metastatic stratum (top 10 features for each primary site displayed in supplementary information S7), enabling discrimination between the organs of origin of the metastatic cancer (lung vs. melanoma vs. breast), focusing upon secondary brain tumours are originating from vibrations of C–O, C=O and C–H associated with lipids and protein macromolecules, contributions associated with nucleic material (DNA/RNA via PO_2_^−^) and minimal contributions from the Amide spectral regions. This correlates with research performed by Gazi et al. [23, 24] when utilizing FTIR microscopy to investigate discrimination of metastatic prostate cancer tissue and organ confined prostate cancer. Gazi et al. show increases in biomolecular intensities of carbohydrate, phosphate and lipid hydrocarbon intensities between organ confined prostate cancer and prostate cancer bone metastases tissue specimens. Krafft et al. highlight spectral features at 1026, 1080 and 1153 cm^−1^ as molecular markers for brain metastases of the primary tumour renal cell carcinoma, the intensity at 1735 cm^−1^, assigned to the carbonyl vibrations (C=O) of ester groups as indicative of brain metastases of breast cancer, an increase in Amide II intensity and broadening of the Amide I low wavenumber shoulder near 1625 cm^−1^ for brain metastases of lung cancer and an intensity minimum near 1400 cm^−1^ for brain metastases of colorectal cancer when performing IR spectroscopic imaging of brain tissue [25]. The similar regions observed for the tissue spectroscopic studies as compared to serum based spectroscopic studies provide corroborating evidence or the power of the analysis as the serum biochemical profile is understood to reflect the tissue status.

In order to examine the ability of feature extraction to improve the diagnostic capability of stratified serum diagnostics a 525 iteration feature-fed SVM was performed using all of the 130 features discovered during the feature extraction process, the top 30 features and the top 2 features for the cancer versus non-cancer stratum, based on a variable ranking process. All 130 features are displayed in supplementary information Table S8. Highlighting the spectral regions described previously.

Supplementary information S9 displays the histograms showing the sensitivity and specificities achieved when analysing 525 iterations of a 130 feature-fed SVM (A), 30 feature-fed SVM (B) and 2 feature-fed SVM (C) for the cancer versus non-cancer stratum. When compared to the full fingerprint region SVM shown in supplementary information S3 the range of sensitivities and specificities observed achieve higher percentages and occur over a smaller range, when compared to the SVM analysis of data from the full spectral fingerprint region, from 81 to 97 % and 51–95 % respectively for -the fingerprint region SVM and from 82 to 98 % and 66–97 % respectively for the 130 feature-fed SVM, 81–98 % and 66–95 % for the top 30 feature-fed SVM respectively and 81–96 % and 51–95 % for the top 2 feature-fed SVM respectively.

The mode sensitivity and specificity for the full fingerprint region SVM of the cancer versus non-cancer stratum was 89.4 and 78.0 % respectively compared to mode sensitivities and specificities of 92.3 and 80.5 % when using 130 spectral features. The top 30 features achieved 91.3 and 82.9 % when using 30 features and 89.4 and 70.7 % when using 2 spectral features (Table 4). The mean sensitivity and specificity for the feature extracted models follows the same trend with all 130 features achieving 91.5 % sensitivity and 83.0 % specificity, 30 features achieving 90.6 % sensitivity and 81.9 % specificity and 2 features achieving 88.7 % sensitivity and 77.7 % specificity. The mean sensitivities and specificities achieved using full fingerprint region SVM are similar to those that can be achieved using the top 2 spectral features of 89.8 % sensitivity and 77.5 % specificity. The top 2 spectral features that describe the differences between the cancer versus non-cancer disease groupings are RMS energy of C-O groups, PO_2_^−^, RNA/DNA (1176–1242 cm^−1^) versus vibrations PO_2_^−^ stretch of nucleic acids, RNA/DNA (1020–1115 cm^−1^) and the skew of the C-O groups, PO_2_^−^, RNA/DNA (1176–1242 cm^−1^) versus the CH_2_ of lipids/proteins and Amide II (1483–1537 cm^−1^) [18–27].

**Table 4:** Optimum, mean and mode sensitivities and specificities for the cancer versus non-cancer stratum using 130, 30 and 2 spectral features

Model	Optimum sensitivity (%)	Optimum specificity (%)	Mean sensitivity (%)	Mean specificity (%)	Mode sensitivity (%)	Mode specificity (%)
All 130 features	98.1	97.6	91.5	83.0	92.3	80.5
Top 30 features	98.1	95.1	90.6	81.9	91.3	82.9
Top 2 features	96.2	95.1	88.7	77.7	89.4	70.7

We achieved the optimum sensitivities and specificities from our model consisting of all 130 spectral features for cancer versus non-cancer. Features are ranked in order of how representative they are of the original data, thus a reduction in the diagnostic ability from 2 spectral features, compared to all 130 or top 30, is not surprising due to the reduction in spectral information available during feature-fed-SVM.

The ability to select and rank spectral features enables the extraction of data that describes the differences within the disease groupings without addition of added variance based upon other contributing factors from the patients and enables biochemical differences, via spectral peaks, to be observed whereas a full spectral SVM does not. In addition, the selection of spectral features, based upon the collection of the full FTIR spectrum, allows for targeting of the most discriminatory regions during a sparse frequency collection approach [28, 29], and reduction in the processing power required for classification of disease states providing a quicker and more efficient spectroscopic diagnostic process.

### Clinical impact

Vibrational spectroscopy can provide rapid, label-free and objective analysis for clinical practice [26, 27]. This proof of principle project provides substantial translational laboratory research to enable the development of clinical serum spectroscopic diagnostics. The rapidity, ease-of-use, low sample volume, reproducibility and detection characteristics shown by this methodology would provide for a rapid and responsive diagnostic tool that can be used throughout the patient pathway [28]. As such the potential clinical impact of serum spectroscopic diagnostics for brain tumours can be:

1. Robust, rapid diagnostic test with high sensitivity and specificity that can distinguish brain tumours from non cancerous disease prompting more timely onward referral of patients for further testing
2. A test capable of monitoring response to treatment (surgery, radiotherapy, chemotherapy) and detection of recurrent disease enabling serial sample and testing with less cost, resource and radiation exposure compared to conventional methods. In addition such a test may overcome the time lag required to observe changes in tumour size and characteristics on MRI.

### Kappa values

**Fig. 1:**
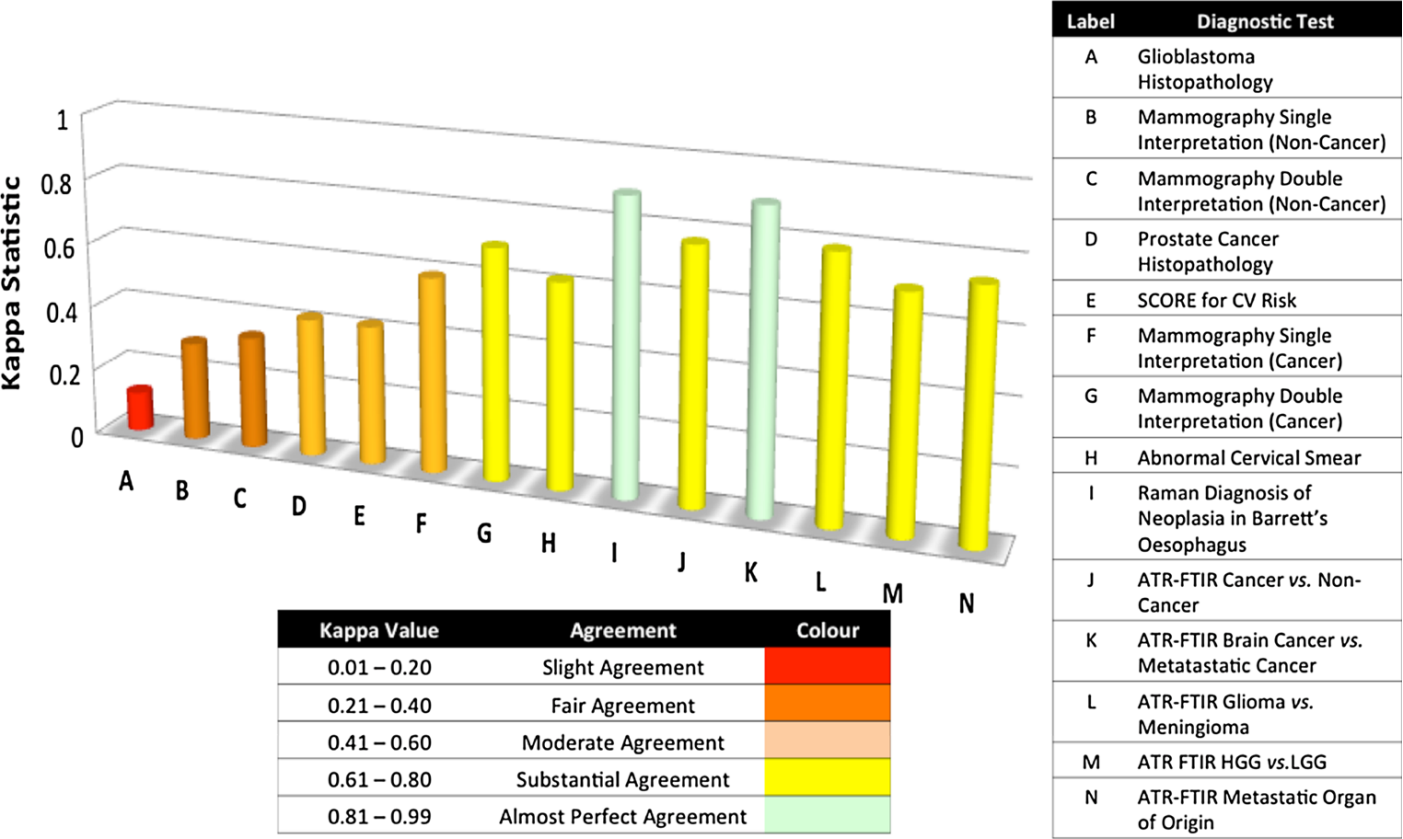
Kappa values for a range of currently used diagnostic tests and proposed spectroscopic diagnoses (A) comparing the histological diagnosis of glioblastoma between local, institutional and central neuro-oncopathology reporting, (B and C) mean Kappa values for breast mammograms using single and double interpretations for non-cancer diagnosis, (D) correlation between Gleason score on biopsy and following prostatectomy, (E) correlation between two commonly used CV risk algorithms Framingham Risk Score (FRS) and European Systemic Coronary Risk Evaluation System (SCORE) compared, (F and G) mean Kappa values for breast mammograms using single and double interpretations for cancer diagnosis, (H) peer review of abnormal cervical smears, (I) Raman spectral prediction of Barrett’s neoplasia in vitro compared to consensus pathology opinion (n = 3 pathologists), (J-N) Kappa values for ATR-FTIR spectroscopic diagnosis based upon optimum sensitivity models over all strata when comparing against clinical diagnosis following multidisciplinary team (MDT) meeting

In order to understand the reliability of a diagnostic model the Kappa value is used to assess the inter-observer agreement whilst correcting for chance (see Materials and Methods), where a Kappa value of <0 indicates a less than chance agreement, 0.01–0.20 slight agreement, 0.21–0.40 fair agreement, 0.41–0.60 moderate agreement, 0.61–0.80 substantial agreement and 0.8–1.00 almost perfect agreement [30]. Figure 1 shows Kappa values from a range of currently used diagnostic tests and proposed spectroscopic diagnoses.

Figure 1 shows a Kappa value of 0.12(A) when comparing the histopathological diagnosis of glioblastoma of 34 patients between local, institutional and central neuro-oncopathology reporting concluding that concordance was sub-optimal when comparing local and central review, however the Kappa value did increase to moderate agreement (k = 0.51) when comparing institutional and central review [31]. For mammography(B, D, F, G) a review of 31 community radiologists concerning 30 women with cancer and 83 without was undertaken to assess the advantages of single versus double interpretation comparing the Kappa values from 465 pairs of radiologist and 31,465 pairs of unique pairs. The mean Kappa values for identify non-cancer radiologist when diagnosing non-cancer was 0.30(B) for single interpretation increasing to 0.34(C) on double interpretation and for cancer was 0.59(F) for single interpretation increasing to 0.70(G) for double interpretation [32]. The correlation between Gleason score at biopsy and prostatectomy of 371 patients undergoing radical prostatectomy revealed a Kappa value of 0.42(D) based upon prostate cancer histopathology concluding that this concordance lies within classical clinical standards [33] and a peer review assessment of 1086 abnormal cervical smears evaluating laboratory cytology performance achieved an overall Kappa value of 0.62(H) when assessing 10 cytologists diagnoses [34]. The Kappa values above are derived from tests that require interpretation from tissue architecture or other diagnostic markers showing a range of Kappa values from 0.12 to 0.70 for these currently used diagnostic tests. It is also interesting to consider a risk factor based test that is performed within the primary care centre in order to direct future treatment and patient care. Examples of such measures are the Framingham Risk Score (FRS) and the European Systemic Coronary Risk Evaluation (SCORE) system for assessing high cardiovascular risk. FRS is widely used within the USA and SCORE is widely used throughout Europe, when comparing the diagnosis of SCORE against that of FRS a Kappa value of 0.42 equating to moderate agreement was achieved [35]. As can be seen from this literature analysis there exists a range of Kappa values from slight agreement to substantial agreement for currently used diagnostic procedures. Kendall et al. used Raman spectroscopy to identify and classify neoplasia in Barrett’s oesophagus when analysing tissue in vitro, in a study utilizing three pathologists to provide a consensus opinion the Kappa value using Raman spectroscopy achieved 0.89(I) [36]. The Kappa values for the ATR-FTIR (J-N) stratified serum diagnostic tests show similar high levels of agreement when comparing against the diagnosis provided following a multidisciplinary team meeting. For cancer versus non-cancer(J) Kappa = 0.77, metastatic versus brain cancer(K) Kappa = 0.90, glioma versus meningioma(L) Kappa = 0.79, high grade Glioma versus low grade Glioma (M) Kappa = 0.70 and the average metastatic model(N) Kappa = 0.74 (lung Kappa = 0.81, skin Kappa = 0.67 and breast Kappa = 0.75). All strata within the stratified serum diagnostics approach showed Kappa values in the substantial and almost perfect agreement ranges.

## Conclusions

The stratified diagnostic methodology discussed has the potential to be intertwined with current healthcare protocols to benefit patient outcomes through early cancer diagnosis. Blood is routinely collected from patients for diagnostic and monitoring purposes, creating no requirement for dedicated sample collection for objective spectral diagnoses. Serum ATR-FTIR spectroscopy involves no sample preparation and is cost-effective due to the minimal use of consumables (to remove the dried serum film from IRE), thus it is a beneficial diagnostic tool with little financial burden.

Rapid stratified serum diagnostics enables the diagnosis of cancer depending on the information required by different multiple clinical settings from a single sample. Using only 1 µl of human serum, a 433 patient dataset (3897 spectra) and collecting spectra within 10 min from serum application to the ATR crystal, we have successfully discriminated, for the first time, between cancer versus non-cancer, cancer severity and the origin of metastatic disease from serum with high sensitivities and specificities. In addition, the feature extraction performed has identified the salient spectral information, reduced patient variance and allows for targeting the most discriminatory regions during spectral collection, thus reducing collection times. This research examines the ability of feature extraction to improve diagnostic ability by extracting discriminatory features of the original spectral data. The proposed stratified diagnostic approach has substantial and almost perfect inter-observer agreement Kappa values, supporting the use of our diagnostic models in a clinical setting. We believe the ability to reduce the time to diagnosis based upon a relatively non-invasive diagnostic test, with significant inter-observer agreement and one that is capable of deployment across clinical situations (dependent upon the diagnostic question posed) would provide rapid patient entry to the clinical process, profiling of at-risk population cohorts, as well as enabling close clinical follow up throughout resulting in a reduction in mortality and morbidity and increases healthcare efficiency.

## Electronic supplementary material

Below is the link to the electronic supplementary material.

Supplementary material 1 (DOC 7738 kb)
