# Supplementary material for: Brain tumour differentiation: rapid stratified serum diagnostics via attenuated total reflection Fourier-transform infrared spectroscopy
Source: J Neurooncol. 2016 Feb 13;127:463–72. doi: 10.1007/s11060-016-2060-x (PMC4835510; doi:10.1007/s11060-016-2060-x)
Supplement: Supplementary file 1 — Supplementary material 1 (DOC 7738 kb) [file 11060_2016_2060_MOESM1_ESM.doc]

**Supplementary Information**

**
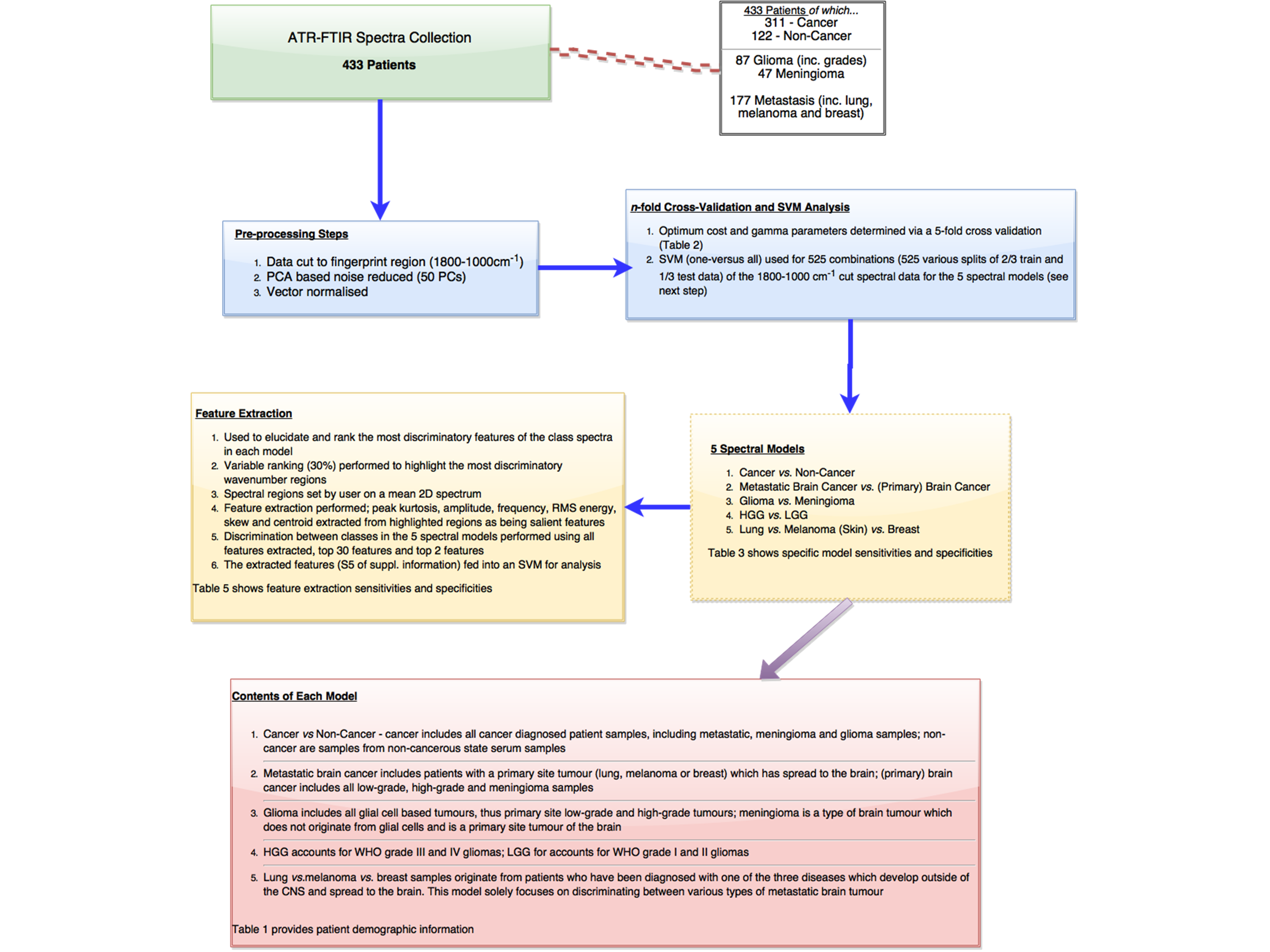
**

**S1.** Flow diagram showing analysis and pre-processing steps, including further detail on which patient samples are included in which classification set.

**S2.** Table describing full patient information **(**patient number, gender, age at sample collection and diagnosis) for all patients used in the study

| **Patient Number** | **Gender** | **Patient Age at Sample Collection** | | | **Diagnosis** | |
| --- | --- | --- | --- | --- | --- | --- |
| **Non-Cancer Patients** |  |  | | |  | |
| **574** | F | 26 | | | Normal | |
| **575** | F | 67 | | | Normal | |
| **576** | M | 81 | | | Normal | |
| **579** | F | 61 | | | Normal | |
| **580** | F | 80 | | | Normal | |
| **582** | F | 74 | | | Normal | |
| **583** | M | 39 | | | Normal | |
| **585** | M | 34 | | | Normal | |
| **586** | M | 60 | | | Normal | |
| **587** | F | 65 | | | Normal | |
| **589** | M | 45 | | | Normal | |
| **591** | M | 85 | | | Normal | |
| **592** | F | 67 | | | Normal | |
| **593** | M | 84 | | | Normal | |
| **594** | M | 57 | | | Normal | |
| **595** | M | 39 | | | Normal | |
| **598** | F | 68 | | | Normal | |
| **600** | M | 68 | | | Normal | |
| **603** | M | 87 | | | Normal | |
| **606** | M | 37 | | | Normal | |
| **607** | F | 56 | | | Normal | |
| **608** | F | 47 | | | Normal | |
| **609** | M | 55 | | | Normal | |
| **610** | M | 57 | | | Normal | |
| **616** | M | 65 | | | Normal | |
| **933** | M | 41 | | | Normal | |
| **1115** | M | 89 | | | Normal | |
| **1116** | F | 64 | | | Normal | |
| **1127** | F | 44 | | | Normal | |
| **1128** | F | 43 | | | Normal | |
| **1133** | M | 76 | | | Normal | |
| **1136** | F | 72 | | | Normal | |
| **1367** | F | 34 | | | Normal | |
| **1368** | M | 46 | | | Normal | |
| **1369** | F | 36 | | | Normal | |
| **1370** | F | 52 | | | Normal | |
| **1371** | F | 49 | | | Normal | |
| **1372** | F | 41 | | | Normal | |
| **1373** | F | 41 | | | Normal | |
| **1374** | F | 40 | | | Normal | |
| **1375** | M | 23 | | | Normal | |
| **1376** | M | 53 | | | Normal | |
| **1390** | M | 70 | | | Normal | |
| **1391** | M | 71 | | | Normal | |
| **1392** | M | 34 | | | Normal | |
| **1393** | F | 71 | | | Normal | |
| **1394** | M | 57 | | | Normal | |
| **1395** | M | 66 | | | Normal | |
| **1396** | M | 21 | | | Normal | |
| **1397** | M | 32 | | | Normal | |
| **1398** | M | 40 | | | Normal | |
| **1399** | F | 24 | | | Normal | |
| **1400** | M | 39 | | | Normal | |
| **1401** | M | 65 | | | Normal | |
| **1402** | F | 25 | | | Normal | |
| **1403** | F | 67 | | | Normal | |
| **1475** | M | 22 | | | Normal | |
| **1476** | M | 41 | | | Normal | |
| **1477** | F | 50 | | | Normal | |
| **1478** | M | 47 | | | Normal | |
| **1479** | M | 16 | | | Normal | |
| **1480** | F | 39 | | | Normal | |
| **1481** | F | 28 | | | Normal | |
| **1482** | M | 52 | | | Normal | |
| **1483** | F | 53 | | | Normal | |
| **1484** | M | 56 | | | Normal | |
| **1485** | M | 51 | | | Normal | |
| **1486** | M | 56 | | | Normal | |
| **1487** | F | 53 | | | Normal | |
| **1488** | M | 54 | | | Normal | |
| **1489** | F | 49 | | | Normal | |
| **1490** | F | 23 | | | Normal | |
| **1491** | F | 31 | | | Normal | |
| **1492** | M | 31 | | | Normal | |
| **1493** | M | 24 | | | Normal | |
| **1494** | M | 31 | | | Normal | |
| **1495** | F | 33 | | | Normal | |
| **1496** | F | 37 | | | Normal | |
| **1497** | M | 47 | | | Normal | |
| **1498** | F | 62 | | | Normal | |
| **1499** | M | unknown | | | Normal | |
| **1500** | F | unknown | | | Normal | |
| **1501** | M | 49 | | | Normal | |
| **1502** | M | 28 | | | Normal | |
| **1503** | M | 27 | | | Normal | |
| **1504** | M | 28 | | | Normal | |
| **1505** | F | 32 | | | Normal | |
| **1506** | F | 32 | | | Normal | |
| **1507** | F | 58 | | | Normal | |
| **1508** | F | 51 | | | Normal | |
| **1509** | F | 24 | | | Normal | |
| **1510** | M | 58 | | | Normal | |
| **1511** | unknown | 57 | | | Normal | |
| **1512** | M | 25 | | | Normal | |
| **1513** | M | 56 | | | Normal | |
| **1514** | F | 25 | | | Normal | |
| **1515** | F | 57 | | | Normal | |
| **1516** | F | 43 | | | Normal | |
| **1517** | F | 51 | | | Normal | |
| **1518** | M | 32 | | | Normal | |
| **1519** | F | 35 | | | Normal | |
| **1520** | F | 27 | | | Normal | |
| **1521** | M | 49 | | | Normal | |
| **1522** | M | unknown | | | Normal | |
| **1523** | M | unknown | | | Normal | |
| **1524** | M | 54 | | | Normal | |
| **1525** | F | 33 | | | Normal | |
| **1526** | M | 29 | | | Normal | |
| **1527** | F | 60 | | | Normal | |
| **1528** | M | 52 | | | Normal | |
| **1529** | F | 51 | | | Normal | |
| **1530** | unknown | 24 | | | Normal | |
| **1531** | M | 25 | | | Normal | |
| **1533** | F | 53 | | | Normal | |
| **1534** | M | 57 | | | Normal | |
| **1535** | M | 29 | | | Normal | |
| **1536** | M | 23 | | | Normal | |
| **1537** | M | 24 | | | Normal | |
| **1538** | F | 23 | | | Normal | |
| **1539** | F | 50 | | | Normal | |
| **1540** | F | 25 | | | Normal | |
| **1541** | F | 50 | | | Normal | |
| **Low-Grade Glioma Patients** |  |  | | |  | |
| **932** | F | 59 | | | Oligoastrocytoma | |
| **1043** | M | 21 | | | Ganglioglioma | |
| **1044** | M | 29 | | | Infiltrating edge of glioma | |
| **1046** | F | 19 | | | LGG diagnosis only | |
| **1047** | F | 54 | | | Ependymoma | |
| **1048** | F | 35 | | | LGG diagnosis only | |
| **1049** | M | 29 | | | LGG diagnosis only | |
| **11006** | F | 34 | | | Astrocytoma | |
| **11017** | M | 40 | | | Astrocytoma | |
| **11020** | M | 50 | | | Astrocytoma | |
| **11084** | F | 30 | | | Astrocytoma | |
| **11109** | M | 46 | | | Oligodendroglioma | |
| **12001** | F | 60 | | | Astrocytoma | |
| **12060** | F | 53 | | | Oligoastrocytoma | |
| **12100** | M | 37 | | | Oligoastrocytoma | |
| **12103** | F | 31 | | | Oligodendroglioma | |
| **12118** | M | 20 | | | Astrocytoma | |
| **12121** | M | 59 | | | Oligodendroglioma | |
| **12136** | M | 41 | | | Oligodendroglioma | |
| **12156** | F | 44 | | | Astrocytoma | |
| **12164** | F | 20 | | | Astrocytoma | |
| **12324** | M | 43 | | | Oligodendroglioma | |
| **12329** | F | 28 | | | Oligoastrocytoma | |
| **High-Grade Glioma Patients** |  |  | | |  | |
| **935** | F | 61 | | | Glioblastoma multiforme | |
| **1110** | M | 33 | | | Glioblastoma multiforme | |
| **1138** | M | 40 | | | Oligoastrocytoma III | |
| **1150** | M | 57 | | | Gliosarcoma IV | |
| **1175** | M | 75 | | | Glioblastoma multiforme | |
| **1314** | M | 70 | | | Glioma III | |
| **11001** | M | 62 | | | Glioblastoma multiforme | |
| **11004** | M | 55 | | | Glioblastoma multiforme | |
| **11005** | M | 66 | | | Glioblastoma multiforme | |
| **11012** | M | 75 | | | Glioblastoma multiforme | |
| **11014** | M | 44 | | | Glioblastoma multiforme | |
| **11026** | M | 73 | | | Glioblastoma multiforme | |
| **11028** | F | 65 | | | Glioblastoma multiforme | |
| **11029** | M | 66 | | | Glioblastoma multiforme | |
| **11030** | F | 50 | | | Glioblastoma multiforme | |
| **11032** | F | 50 | | | Glioblastoma multiforme | |
| **11034** | M | 58 | | | Glioblastoma multiforme | |
| **11050** | F | 72 | | | Glioblastoma multiforme | |
| **11053** | M | 62 | | | Glioblastoma multiforme | |
| **11057** | M | 41 | | | Glioblastoma multiforme | |
| **11073** | F | 63 | | | Glioblastoma multiforme | |
| **11094** | F | 44 | | | Glioblastoma multiforme | |
| **11097** | M | 45 | | | Glioblastoma multiforme | |
| **11101** | F | 69 | | | Glioblastoma multiforme | |
| **11120** | F | 51 | | | Glioblastoma multiforme | |
| **11121** | F | 79 | | | Glioblastoma multiforme | |
| **11123** | M | 70 | | | Glioblastoma multiforme | |
| **11124** | M | 75 | | | Glioblastoma multiforme | |
| **11137** | F | 66 | | | Glioblastoma multiforme | |
| **11142** | M | 76 | | | Glioblastoma multiforme | |
| **11145** | M | 55 | | | Glioblastoma multiforme | |
| **11154** | M | 67 | | | Glioblastoma multiforme | |
| **11155** | F | 70 | | | Glioblastoma multiforme | |
| **11159** | M | 57 | | | Glioblastoma multiforme | |
| **11163** | M | 68 | | | Glioblastoma multiforme | |
| **11181** | M | 68 | | | Glioblastoma multiforme | |
| **11188** | M | 70 | | | Glioblastoma multiforme | |
| **11197** | F | 61 | | | Glioblastoma multiforme | |
| **11205** | M | 73 | | | Glioblastoma multiforme | |
| **11212** | F | 65 | | | Glioblastoma multiforme | |
| **11268** | F | 74 | | | Glioblastoma multiforme | |
| **12009** | M | 50 | | | Glioblastoma multiforme | |
| **12012** | M | 25 | | | Glioblastoma multiforme | |
| **12014** | F | 64 | | | Glioblastoma multiforme | |
| **12019** | F | 39 | | | Glioblastoma multiforme | |
| **12025** | M | 41 | | | Glioblastoma multiforme | |
| **12028** | F | 72 | | | Glioblastoma multiforme | |
| **12030** | F | 69 | | | Glioblastoma multiforme | |
| **12032** | M | 64 | | | Glioblastoma multiforme | |
| **12042** | F | 55 | | | Glioblastoma multiforme | |
| **12046** | F | 53 | | | Glioblastoma multiforme | |
| **12054** | M | 65 | | | Glioblastoma multiforme | |
| **12055** | M | 25 | | | Glioblastoma multiforme | |
| **12057** | M | 66 | | | Glioblastoma multiforme | |
| **12063** | M | 56 | | | Glioblastoma multiforme | |
| **451** | M | 75 | | | Glioblastoma multiforme | |
| **499** | M | 73 | | | Glioblastoma multiforme | |
| **549** | F | 67 | | | Glioblastoma multiforme | |
| **556** | F | 81 | | | Glioblastoma multiforme | |
| **615** | M | 68 | | | Glioblastoma multiforme | |
| **733** | M | 74 | | | Glioblastoma multiforme | |
| **763** | M | 67 | | | Glioblastoma multiforme | |
| **774** | M | 72 | | | Glioblastoma multiforme | |
| **769** | M | 70 | | | Glioblastoma multiforme | |
| **Meningioma Patients** |  |  | | |  | |
| **11066** | F | 68 | | | Meningioma | |
| **11068** | F | 71 | | | Meningioma | |
| **11070** | F | 56 | | | Meningioma | |
| **11090** | F | 34 | | | Meningioma | |
| **11111** | M | 60 | | | Meningioma | |
| **11127** | F | 49 | | | Meningioma | |
| **11173** | F | 69 | | | Meningioma | |
| **11191** | M | 69 | | | Meningioma | |
| **11215** | F | 44 | | | Meningioma | |
| **11214** | F | 69 | | | Meningioma | |
| **12043** | M | 49 | | | Meningioma | |
| **12056** | F | 57 | | | Meningioma | |
| **12058** | M | 73 | | | Meningioma | |
| **12077** | F | 67 | | | Meningioma | |
| **12085** | F | 58 | | | Meningioma | |
| **12115** | F | 64 | | | Meningioma | |
| **12127** | F | 61 | | | Meningioma | |
| **12157** | F | 73 | | | Meningioma | |
| **12163** | F | 66 | | | Meningioma | |
| **12261** | F | 68 | | | Meningioma | |
| **12326** | M | 39 | | | Meningioma | |
| **12396** | F | 59 | | | Meningioma | |
| **12418** | F | 67 | | | Meningioma | |
| **12441** | M | 53 | | | Meningioma | |
| **12449** | M | 51 | | | Meningioma | |
| **12452** | M | 75 | | | Meningioma | |
| **12470** | F | 62 | | | Meningioma | |
| **13028** | F | 43 | | | Meningioma | |
| **13039** | F | 35 | | | Meningioma | |
| **13059** | F | 50 | | | Meningioma | |
| **13062** | F | 72 | | | Meningioma | |
| **13066** | M | 37 | | | Meningioma | |
| **13067** | F | 70 | | | Meningioma | |
| **13077** | F | 41 | | | Meningioma | |
| **13080** | F | 44 | | | Meningioma | |
| **13089** | F | 59 | | | Meningioma | |
| **13090** | F | 48 | | | Meningioma | |
| **13095** | F | 27 | | | Meningioma | |
| **13099** | F | 37 | | | Meningioma | |
| **13112** | M | 64 | | | Meningioma | |
| **13121** | M | 78 | | | Meningioma | |
| **13126** | F | 35 | | | Meningioma | |
| **13133** | F | 78 | | | Meningioma | |
| **13140** | M | 24 | | | Meningioma | |
| **13158** | M | 44 | | | Meningioma | |
| **13162** | F | 48 | | | Meningioma | |
| **13212** | F | 66 | | | Meningioma | |
| **Metastatic Patients** |  |  | | | **Organ of Origin** | |
| **358** | M | 74 | | | Melanoma | |
| **456** | M | 70 | | | Renal | |
| **509** | M | 80 | | | Melanoma | |
| **517** | F | 54 | | | Lung | |
| **553** | F | 72 | | | Renal | |
| **560** | F | 43 | | | Breast | |
| **562** | F | 57 | | | Lung | |
| **567** | F | 63 | | | Melanoma | |
| **666** | F | 61 | | | Lung | |
| **694** | M | 59 | | | Lung | |
| **697** | F | 56 | | | Renal | |
| **700** | F | 50 | | | Colorectal | |
| **712** | F | 39 | | | Breast | |
| **722** | F | 63 | | | Breast | |
| **725** | F | 39 | | | Breast | |
| **745** | M | 50 | | | Lung | |
| **756** | F | 54 | | | Breast | |
| **795** | F | 58 | | | Bowel | |
| **827** | F | 55 | | | Breast | |
| **837** | F | 68 | | | Lung | |
| **841** | F | 36 | | | Melanoma | |
| **847** | M | 63 | | | Lung | |
| **853** | F | 55 | | | Bowel | |
| **865** | F | 63 | | | Lung | |
| **866** | M | 82 | | | Lung | |
| **884** | F | 65 | | | Lung | |
| **888** | M | 63 | | | Colorectal | |
| **893** | M | 75 | | | Lung | |
| **901** | M | 63 | | | Melanoma | |
| **912** | F | 50 | | | Breast | |
| **943** | F | 64 | | | Breast | |
| **947** | F | 64 | | | Lung | |
| **952** | M | 58 | | | Lung | |
| **972** | F | 50 | | | Breast | |
| **1001** | F | 67 | | | Lung | |
| **1011** | F | 68 | | | Lung | |
| **1012** | F | 52 | | | Breast | |
| **1025** | F | 77 | | | Rectal | |
| **1031** | M | 65 | | | Lung | |
| **1040** | F | 51 | | | Breast | |
| **1041** | M | 59 | | | Renal | |
| **1053** | M | 53 | | | Lung | |
| **1056** | F | 54 | | | Melanoma | |
| **1057** | M | 63 | | | Lung | |
| **1060** | M | 63 | | | Lung | |
| **1070** | M | 71 | | | Lung | |
| **1081** | M | 67 | | | Melanoma | |
| **1082** | M | 71 | | | Lung | |
| **1092** | M | 63 | | | Lung | |
| **1103** | F | 39 | | | Lung | |
| **1111** | M | 63 | | | Melanoma | |
| **1113** | F | 69 | | | Lung | |
| **1117** | F | 75 | | | Lung | |
| **1144** | F | 67 | | | Breast | |
| **1148** | F | 67 | | | Breast | |
| **1149** | M | 53 | | | Bowel | |
| **1158** | F | 55 | | | Breast | |
| **1184** | F | 59 | | | Lung | |
| **1311** | F | 74 | | | Lung | |
| **1326** | M | 73 | | | Renal | |
| **1327** | M | 55 | | | Renal | |
| **1330** | F | 77 | | | Lung | |
| **1338** | F | 71 | | | Lung | |
| **1348** | F | 81 | | | Lung | |
| **1354** | F | 40 | | | Lung | |
| **1382** | F | 44 | | | Breast | |
| **1431** | M | 54 | | | Melanoma | |
| **1438** | F | 80 | | | Lung | |
| **1443** | F | 52 | | | Breast | |
| **11011** | F | 42 | | | Melanoma | |
| **11019** | M | 59 | | | Non-small cell lung cancer (NSCLC) Adenocarcinoma | |
| **11027** | F | 64 | | | Breast | |
| **11037** | M | 67 | | | Non-small cell lung cancer (NSCLC) Adenocarcinoma | |
| **11043** | F | 59 | | | Lung | |
| **11047** | F | 67 | | | Lung | |
| **11049** | M | 59 | | | Non-small cell lung cancer (NSCLC) Squamous cell | |
| **11052** | F | 79 | | | Non-small cell lung cancer (NSCLC) Adenocarcinoma | |
| **11054** | F | 61 | | | Non-small cell lung cancer (NSCLC) Adenocarcinoma | |
| **11059** | M | 68 | | | Unknown primary, brain metastasis is adenocarcinoma | |
| **11075** | F | 74 | | | Melanoma | |
| **11076** | F | 60 | | | Lung | |
| **11080** | F | 29 | | | Colon | |
| **11087** | M | 71 | | | Non-small cell lung cancer (NSCLC) Adenocarcinoma | |
| **11089** | M | 73 | | | Non-small cell lung cancer (NSCLC), brain metastasis is large cell neuroendocrine | |
| **11103** | F | 64 | | | Ovarian | |
| **11104** | M | 65 | | | Renal | |
| **11108** | F | 55 | | | Breast | |
| **11110** | M | 66 | | | NSCLC | |
| **11146** | M | 59 | | | Non-small cell lung cancer (NSCLC) Adenocarcinoma | |
| **11152** | F | 44 | | | Breast | |
| **11161** | M | 64 | | | Non-small cell lung cancer (NSCLC) Adenocarcinoma | |
| **11164** | F | 25 | | | Melanoma | |
| **11187** | F | 50 | | | Adenocarcinoma bowel | |
| **11193** | M | 41 | | | Melanoma | |
| **11213** | F | 60 | | | Non-small cell lung cancer (NSCLC) Adenocarcinoma | |
| **12011** | M | 62 | | | Renal | |
| **12037** | F | 52 | | | Lung | |
| **12041** | F | 64 | | | Non-small cell lung cancer (NSCLC) Adenocarcinoma | |
| **12044** | F | 36 | | | Melanoma | |
| **12051** | F | 48 | | | Breast | |
| **12071** | M | 49 | | | Melanoma | |
| **12074** | F | 76 | | | Breast | |
| **12098** | F | 44 | | | Breast | |
| **12104** | F | 59 | | | Non-small cell lung cancer (NSCLC) Adenocarcinoma | |
| **12133** | M | 56 | | | Non-small cell lung cancer (NSCLC) Adenocarcinoma | |
| **12135** | F | 27 | | | Breast | |
| **12138** | F | 44 | | | Breast | |
| **12145** | F | 64 | | | Non-small cell lung cancer (NSCLC) Adenocarcinoma | |
| **12148** | M | 65 | | | Renal | |
| **12149** | M | 56 | | | Non-small cell lung cancer (NSCLC) Adenocarcinoma | |
| **12158** | F | 73 | | | Non-small cell lung cancer (NSCLC) Adenocarcinoma | |
| **12160** | F | 63 | | | Breast | |
| **12166** | F | 74 | | | Non-small cell lung cancer (NSCLC) Adenocarcinoma | |
| **12179** | F | 70 | | | Non-small cell lung cancer (NSCLC) Adenocarcinoma | |
| **12180** | F | 41 | | | Ovarian | |
| **12212** | M | 65 | | | Non-small cell lung cancer (NSCLC) Adenocarcinoma | |
| **12262** | F | 67 | | | Non-small cell lung cancer (NSCLC) Squamous cell | |
| **12264** | F | 69 | | | Non-small cell lung cancer (NSCLC) Adenocarcinoma | |
| **12313** | M | 65 | | | Bowel | |
| **12321** | F | 39 | | | Breast | |
| **12381** | F | 65 | | | Renal | |
| **12382** | F | 58 | | | Non-small cell lung cancer (NSCLC) Adenocarcinoma | |
| **12385** | F | 66 | | | Non-small cell lung cancer (NSCLC) Adenocarcinoma | |
| **12397** | F | 62 | | | Renal | |
| **12403** | M | 66 | | | Melanoma | |
| **12416** | F | 78 | | | Lung | |
| **12438** | F | 40 | | | Breast | |
| **12448** | F | 52 | | | Breast | |
| **12460** | M | 53 | | | Non-small cell lung cancer (NSCLC) Adenocarcinoma | |
| **13010** | F | 65 | | | Non-small cell lung cancer (NSCLC) Adenocarcinoma | |
| **13022** | F | 58 | | | Breast | |
| **13035** | F | 39 | | | Melanoma | |
| **13042** | F | 44 | | | Melanoma | |
| **13050** | F | 66 | | | Non-small cell lung cancer (NSCLC) Adenocarcinoma | |
| **13051** | F | 53 | | | Melanoma | |
| **13060** | M | 74 | | | Non-small cell lung cancer (NSCLC) Adenocarcinoma | |
| **13065** | M | 58 | | | Colon | |
| **13072** | M | 70 | | | Melanoma | |
| **13075** | F | 49 | | | Breast | |
| **13079** | F | 66 | | | Non-small cell lung cancer (NSCLC) Adenocarcinoma | |
| **13101** | M | 26 | | | Testicular | |
| **13107** | M | 66 | | | Non-small cell lung cancer (NSCLC) Adenocarcinoma | |
| **13110** | F | 44 | | | Breast | |
| **13113** | M | 66 | | | Lung | |
| **13130** | F | 57 | | | Breast | |
| **13176** | M | 67 | | | Melanoma | |
| **13196** | M | 60 | | | Non-small cell lung cancer (NSCLC) Adenocarcinoma | |
| **13218** | F | 39 | | | Breast | |
| **13222** | F | unknown | | | Non-small cell lung cancer (NSCLC) | |
| **13226** | | | F | 66 | | Non-small cell lung cancer (NSCLC) |
| **13244** | | | F | 60 | | Non-small cell lung cancer (NSCLC) |
| **13245** | | | F | 62 | | Endomaterial |
| **13272** | | | M | 75 | | Melanoma |
| **13276** | | | F | 67 | | Non-small cell lung cancer (NSCLC) Squamous cell |
| **13281** | | | M | 66 | | Non-small cell lung cancer (NSCLC) Adenocarcinoma |
| **13288** | | | M | 68 | | Non-small cell lung cancer (NSCLC) Adenocarcinoma |
| **13302** | | | F | 35 | | Melanoma |
| **13305** | | | M | 73 | | Melanoma |
| **13306** | | | F | 57 | | Breast |
| **13311** | | | F | 53 | | Lung |
| **13326** | | | M | 46 | | Lung |
| **13401** | | | M | 68 | | Lung |
| **13405** | | | F | 73 | | Lung |
| **13426** | | | F | 39 | | Breast |
| **13431** | | | M | 72 | | Large-cell Neuroendrocrine (Lung) |
| **13492** | | | M | 68 | | Lung |
| **13510** | | | F | 72 | | Colon |
| **13624** | | | F | 69 | | Lung |
| **13626** | | | F | 59 | | Lung |
| **13666** | | | M | 76 | | Lung (Atypical Carcinoid) |
| **13667** | | | M | 38 | | Non-small cell lung cancer (NSCLC) Adenocarcinoma |
| **13668** | | | M | 63 | | Non-small cell lung cancer (NSCLC with Adenocarcimona Oesophagus |
| **13669** | | | M | 69 | | Metastatic carcimona from an unknown primary |
| **13670** | | | F | 49 | | Breast |
| **13671** | | | M | 53 | | Renal |
| **13672** | | | M | 57 | | Melanoma |
| **13673** | | | M | 42 | | Papillary Carcinoma Metastasis (Renal) |

**S3.** Total subject number of tumour grade, age range, mean age and gender of patient samples

|  | Cancer vs. Non-cancer | Metastasis vs. Brain | Glioma vs. Meningioma | HGG vs. LGG | Lung vs. Skin vs. Breast |
| --- | --- | --- | --- | --- | --- |
| Optimal Cost (C) | 128 | 128 | 128 | 128 | 32 |
| Optimal Gamma (*γ*) | 128 | 128 | 128 | 128 | 128 |
| Mean Cross-Validation (CV) Accuracy | 94.74 % | 94.07 % | 95.37 % | 95.60 % | 93.10 % |

**S4.** Histograms showing the sensitivity and specificity results for 525 iterations of SVM conducted using the fingerprint region from 1800-1000 cm^-1^


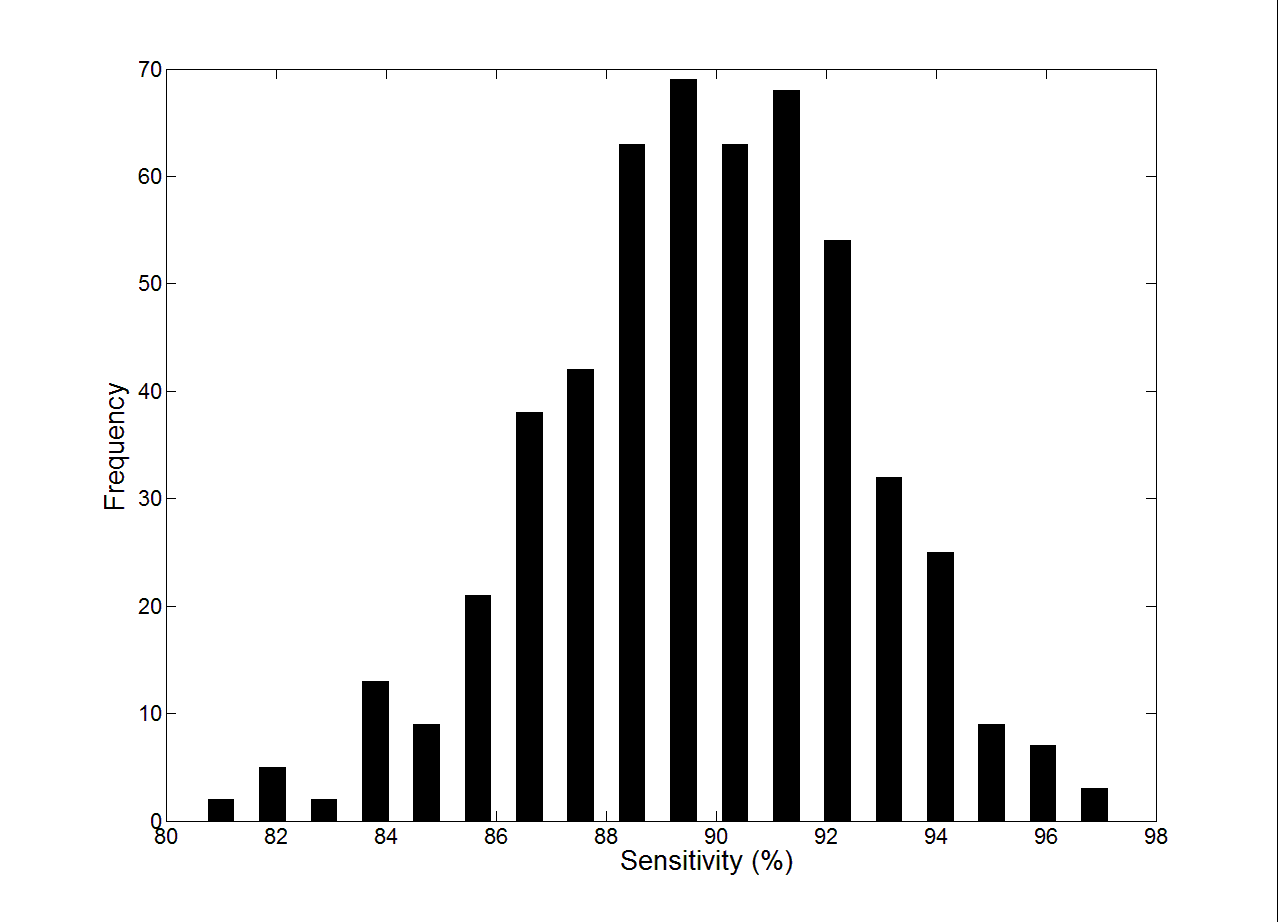

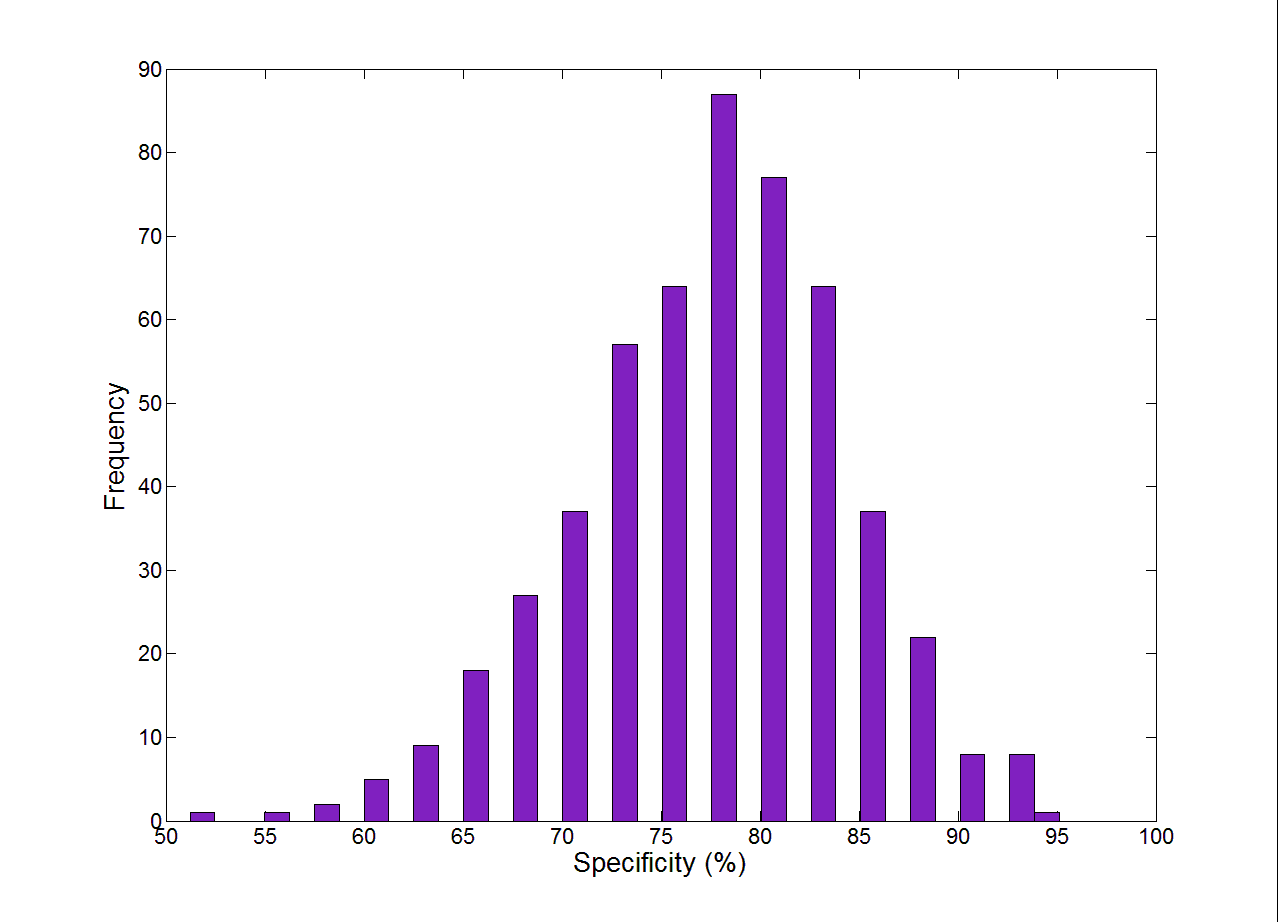


**
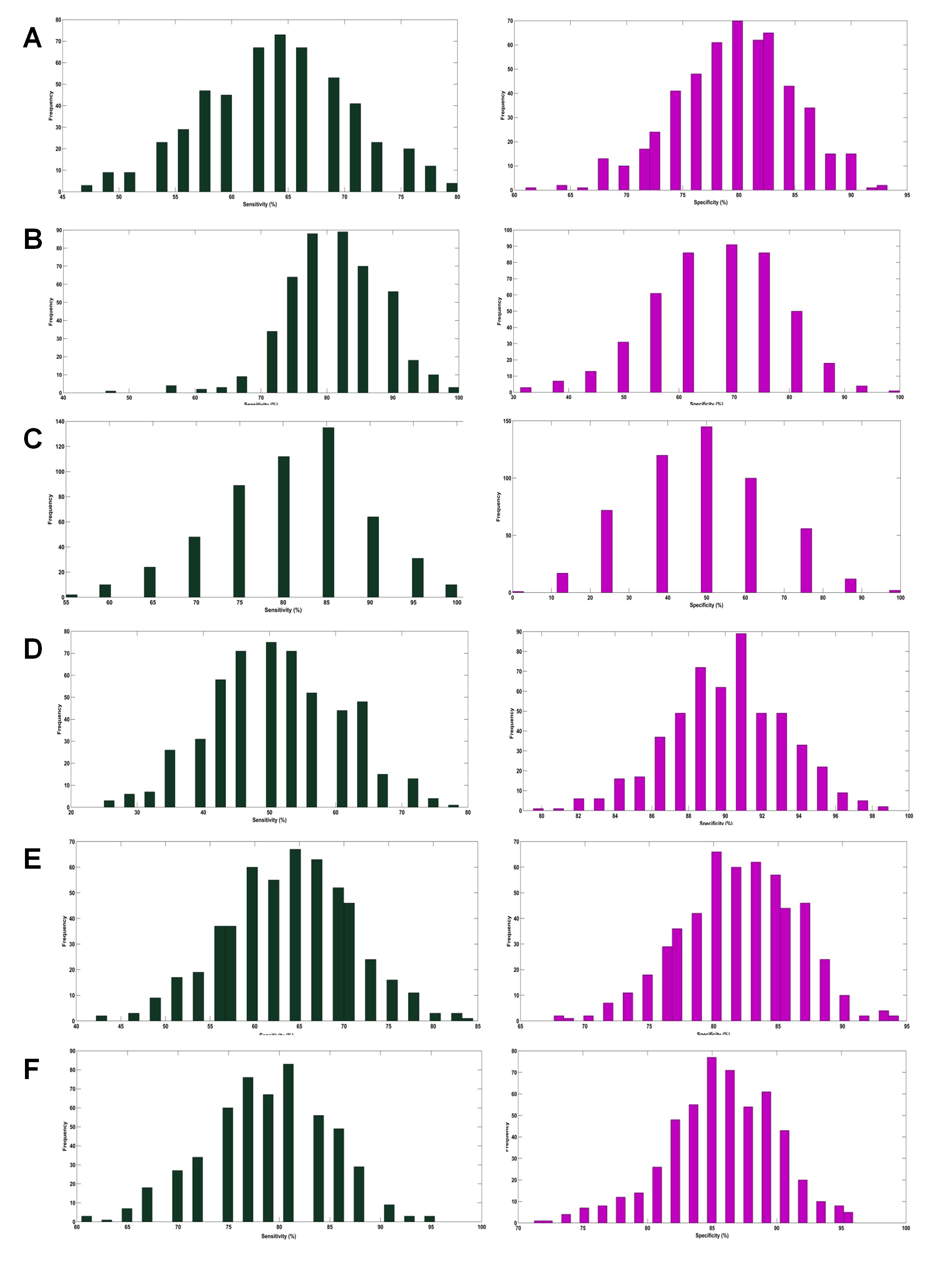
**

**S5.** Histograms showing the sensitivity (black) and specificity (purple) results of 525 spectral data set iterations (2/3 spectra in training set per disease class and 1/3 spectra in blind set per disease class for each disease grouping using a patient based splitting protocol) for (A) Metastatic Cancer vs. Brain Cancer (B) Glioma vs. Meningioma (C) High Grade Glioma (HGG) vs. Low Grade Glioma (LGG) (D) Metastatic Lung vs. Skin vs. Breast Model - Breast Results (E) Metastatic Lung vs. Skin vs. Breast Model - Skin Results (F) Metastatic Lung vs. Skin vs. Breast Model - Lung Results


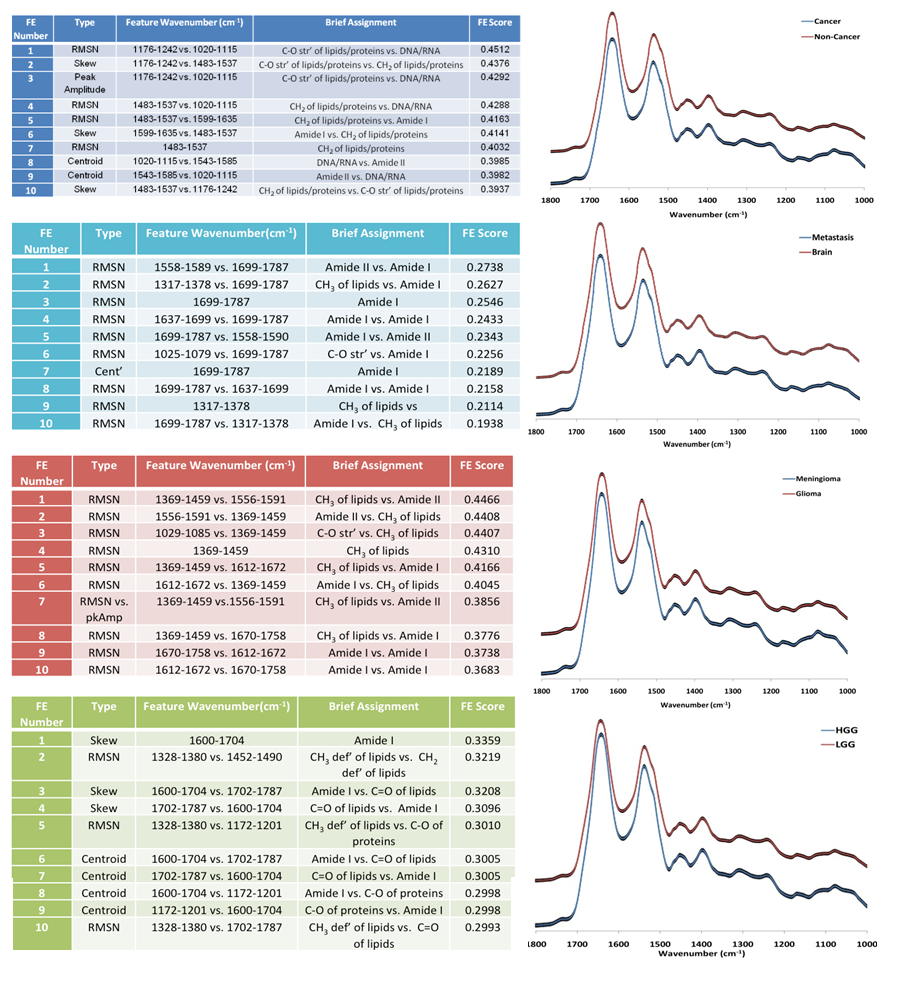


**S6.** Top ten extracted features, based upon feature extraction score, and average spectrum from each group in each strata for (A) Cancer vs. Non-cancer, (B) Metastatic Cancer vs. Brain Cancer, (C) Glioma vs. Meningioma and (D) High Grade Glioma (HGG) vs. Low Grade Glioma (LGG). All spectra (offset for ease of visualisation) display standard deviation error bars presented as a cloud around the spectrum.


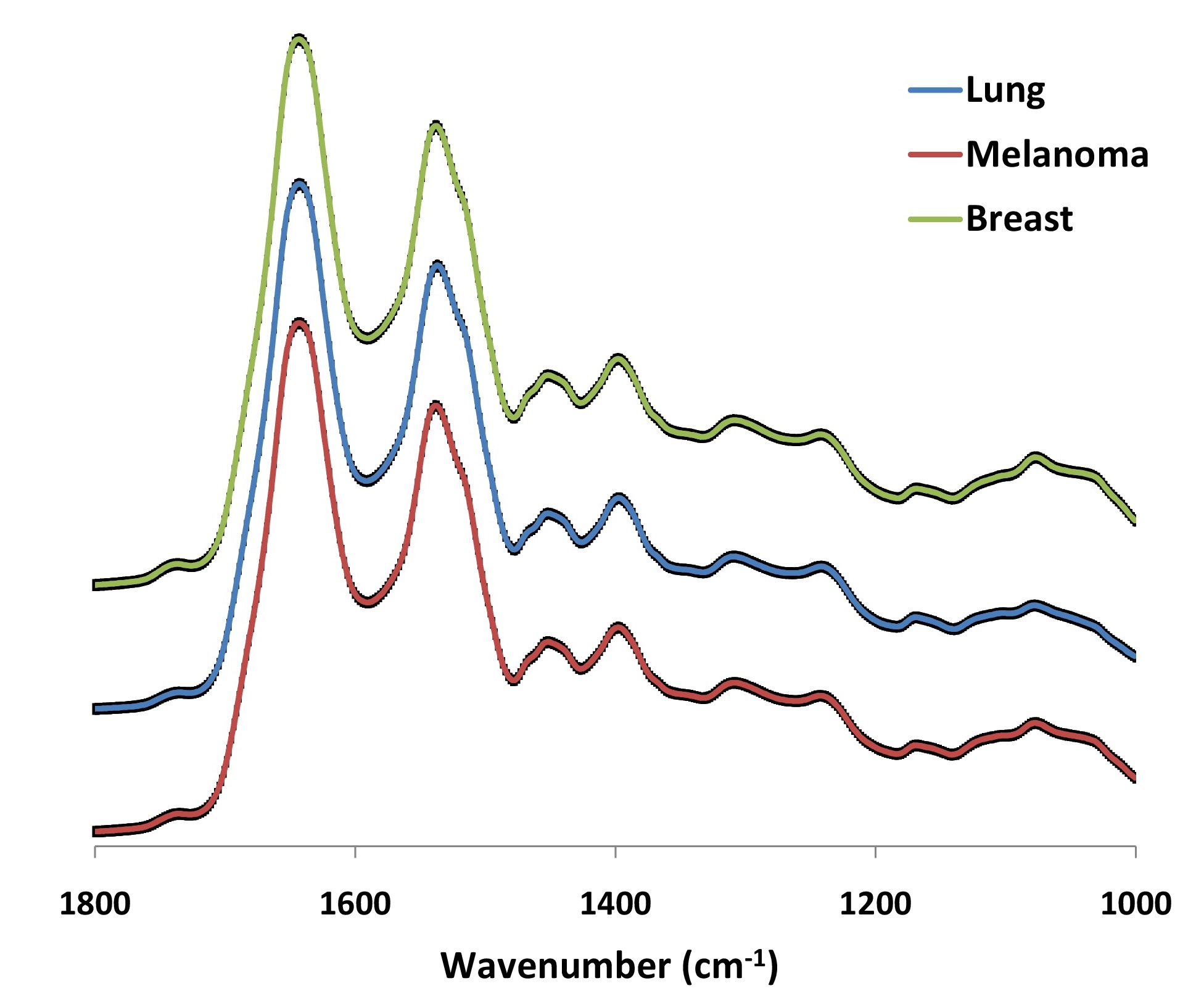

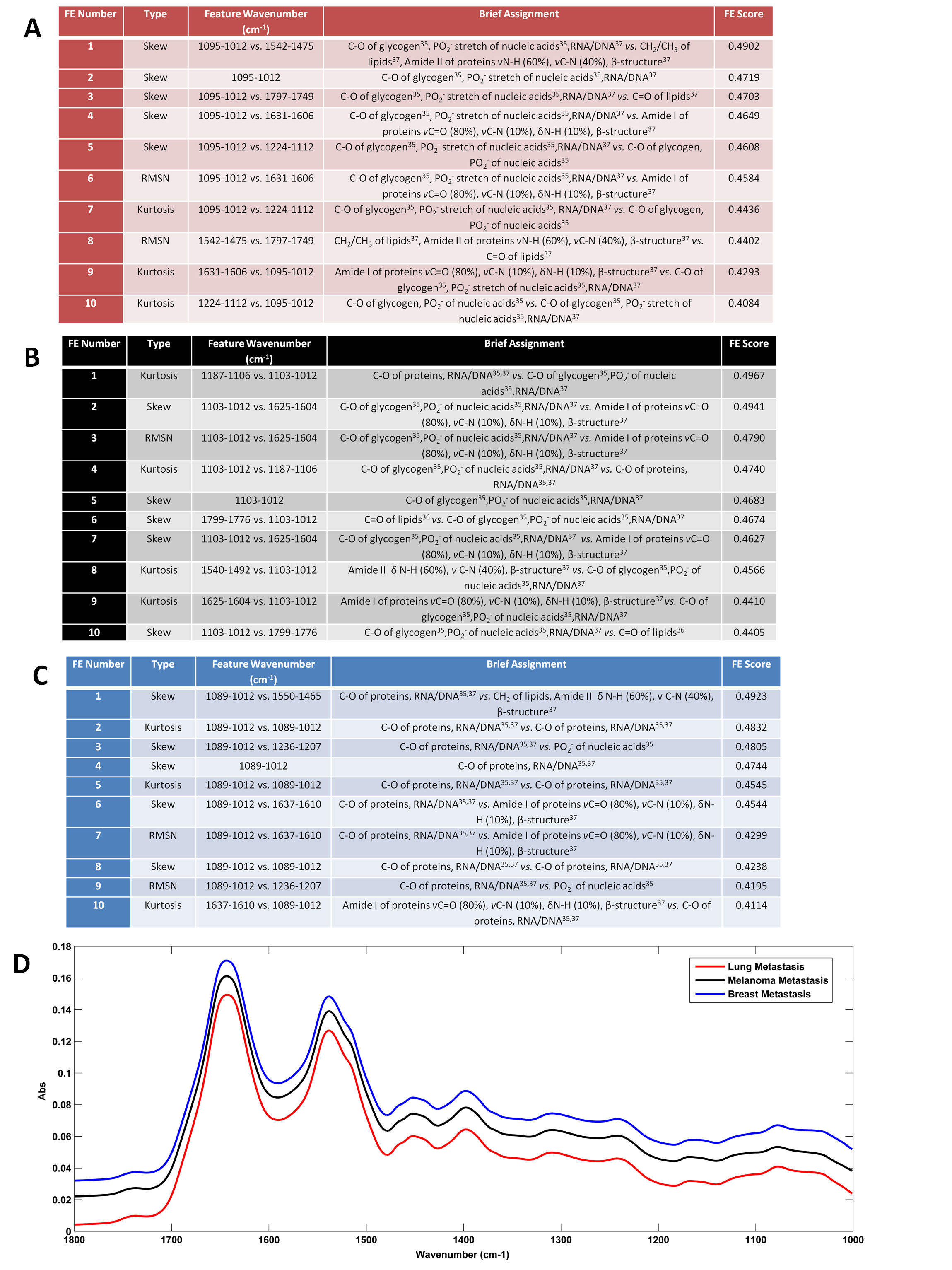


**D**

**S7.** Top ten extracted features, based upon feature extraction score, and average spectrum from each group in the metastatic origin stratum (A) Lung metastases features, (B) Melanoma metastases features, (C) Breast metastases features and (D) average spectra from metastatic serum samples. All spectra (offset for ease of visualisation) display standard deviation error bars presented as a cloud around the spectrum.

**S8.** All 130 spectral features (type and wavenumber) selected by variable ranking from the cancer vs. non-cancer stratum

| FE Number | Type | Actual WN (cm^-1^) | FE Score |
| --- | --- | --- | --- |
| **1** | RMSN | 1176-1242 vs. 1020-1115 | 0.4512 |
| **2** | Skew | 1176-1242 vs. 1483-1537 | 0.4376 |
| **3** | Peak Amplitude | 1176-1242 vs. 1020-1115 | 0.4292 |
| **4** | RMSN | 1483-1537 vs. 1020-1115 | 0.4288 |
| **5** | RMSN | 1483-1537 vs. 1599-1635 | 0.4163 |
| **6** | Skew | 1599-1635 vs. 1483-1537 | 0.4141 |
| **7** | RMSN | 1483-1537 | 0.4032 |
| **8** | Centroid | 1020-1115 vs. 1543-1585 | 0.3985 |
| **9** | Centroid | 1543-1585 vs. 1020-1115 | 0.3982 |
| **10** | Skew | 1483-1537 vs. 1176-1242 | 0.3937 |
| **11** | Centroid | 1020-1115 vs. 1483-1537 | 0.3918 |
| **12** | Centroid | 1483-1537 vs. 1020-1115 | 0.3914 |
| **13** | RMSN | 1599-1635 vs. 1483-1537 | 0.3866 |
| **14** | RMSN | 1020-1115 vs. 1176-1242 | 0.3811 |
| **15** | Centroid | 1020-1115 | 0.3708 |
| **16** | Centroid | 1020-1115 vs. 1641-1693 | 0.3700 |
| **17** | Centroid | 1641-1693 vs. 1020-1115 | 0.3697 |
| **18** | RMSN | 1483-1537 vs. 1543-1585 | 0.3667 |
| **19** | Peak Amplitude | 1483-1537 vs. 1020-1115 | 0.3614 |
| **20** | RMSN | 1020-1115 vs. 1483-1537 | 0.3478 |
| **21** | Peak Amplitude | 1020-1115 vs. 1176-1242 | 0.3473 |
| **22** | Centroid | 1176-1242 vs. 1020-1115 | 0.3445 |
| **23** | Centroid | 1020-1115 vs. 1176-1242 | 0.3443 |
| **24** | Skew | 1543-1585 vs. 1483-1537 | 0.3350 |
| **25** | Skew | 1483-1537 | 0.3325 |
| **26** | Centroid | 1020-1115 vs. 1599-1635 | 0.3313 |
| **27** | Centroid | 1599-1635 vs. 1020-1115 | 0.3312 |
| **28** | Skew | 1020-1115 | 0.3273 |
| **29** | Skew | 1020-1115 vs. 1483-1537 | 0.3258 |
| **30** | Skew | 1020-1115 vs. 1641-1693 | 0.3255 |
| **31** | Peak Amplitude | 1543-1585 vs. 1020-1115 | 0.3243 |
| **32** | Skew | 1641-1693 vs. 1483-1537 | 0.3238 |
| **33** | RMSN | 1543-1585 vs. 1483-1537 | 0.3233 |
| **34** | Peak Amplitude | 1483-1537 vs. 1543-1585 | 0.3226 |
| **35** | Skew | 1020-1115 vs. 1543-1585 | 0.3221 |
| **36** | RMSN | 1020-1115 | 0.3129 |
| **37** | Skew | 1020-1115 vs. 1176-1242 | 0.3117 |
| **38** | Peak Amplitude | 1599-1635 vs. 1020-1115 | 0.3102 |
| **39** | Peak Amplitude | 1543-1585 vs. 1483-1537 | 0.3098 |
| **40** | Centroid | 1599-1635 vs. 1483-1537 | 0.3062 |
| **41** | Centroid | 1483-1537 vs. 1599-1635 | 0.3061 |
| **42** | Peak Amplitude | 1020-1115 vs. 1483-1537 | 0.3008 |
| **43** | RMSN | 1599-1635 vs. 1020-1115 | 0.2916 |
| **44** | Peak Amplitude | 1641-1693 vs. 1020-1115 | 0.2871 |
| **45** | Peak Amplitude | 1020-1115 vs. 1543-1585 | 0.2852 |
| **46** | Peak Amplitude | 1020-1115 | 0.2788 |
| **47** | RMSN | 1020 -1115 vs. 1641-1693 | 0.2783 |
| **48** | Peak Amplitude | 1020 -1115 vs. 1641-1693 | 0.2783 |
| **49** | RMSN | 1543-1585 vs. 1020-1115 | 0.2777 |
| **50** | Peak Amplitude | 1020 -1115 vs. 1599-1635 | 0.2772 |
| **51** | RMSN | 1020 -1115 vs.1543-1585 | 0.2688 |
| **52** | RMSN | 1020 -1115 vs. 1599-1635 | 0.2640 |
| **53** | Centroid | 1599-1635 | 0.2624 |
| **54** | RMSN | 1641-1693 vs. 1020 -1115 | 0.2600 |
| **55** | Skew | 1483-1537 vs. 1543-1585 | 0.2541 |
| **56** | Centroid | 1543-1585 vs. 1599-1635 | 0.2498 |
| **57** | Centroid | 1599-1635 vs. 1543-1585 | 0.2498 |
| **58** | Centroid | 1543-1585 vs. 1641-1693 | 0.2493 |
| **59** | Centroid | 1641-1693 vs.1543-1585 | 0.2493 |
| **60** | RMSN | 1176-1242 vs. 1483-1537 | 0.2466 |
| **61** | RMSN | 1483-1537 vs. 1176-1424 | 0.2432 |
| **62** | Skew | 1483-1537 vs. 1641-1693 | 0.2405 |
| **63** | Skew | 1483-1537 vs. 1599-1635 | 0.2228 |
| **64** | Centroid | 1543-1585 | 0.2212 |
| **65** | RMSN | 1483-1537 vs. 1641-1693 | 0.1827 |
| **66** | Skew | 1020-1115 vs. 1599-1635 | 0.1826 |
| **67** | Kurtosis | 1599-1635 vs. 1176-1242 | 0.1795 |
| **68** | Kurtosis | 1176-1242 vs. 1599-1635 | 0.1733 |
| **69** | Centroid | 1641-1693 vs. 1599-1635 | 0.1730 |
| **70** | Centroid | 1599-1635 vs. 1641-1693 | 0.1730 |
| **71** | RMSN | 1543-1585 vs. 1641-1693 | 0.1719 |
| **72** | Peak Amplitude | 1483-1537 vs. 1599-1635 | 0.1648 |
| **73** | Kurtosis | 1483-1537 vs. 1176-1242 | 0.1638 |
| **74** | Peak Amplitude | 1599-1635 vs. 1483-1537 | 0.1596 |
| **75** | Centroid | 1176-1242 vs. 1599-1635 | 0.1570 |
| **76** | Centroid | 1599-1635 vs. 1176-1242 | 0.1568 |
| **77** | Kurtosis | 1599-1635 vs. 1483-1537 | 0.1558 |
| **78** | RMSN | 1641-1693 vs.1543-1585 | 0.1557 |
| **79** | Centroid | 1641-1693 vs.1483-1537 | 0.1533 |
| **80** | Centroid | 1483-1537 vs. 1641-1693 | 0.1533 |
| **81** | Peak Frequency | 1483-1537 | 0.1525 |
| **82** | Centroid | 1176-1242 vs. 1483-1537 | 0.1484 |
| **83** | Centroid | 1483-1537 vs. 1176-1242 | 0.1483 |
| **84** | Kurtosis | 1483-1537 | 0.1479 |
| **85** | Centroid | 1483-1537 | 0.1400 |
| **86** | RMSN | 1641-1693 vs. 1483-1537 | 0.1372 |
| **87** | Kurtosis | 1543-1585 vs. 1176-1242 | 0.1341 |
| **88** | Peak Frequency | 1020-1115 vs. 1483-1537 | 0.1266 |
| **89** | Peak Amplitude | 1176-1242 vs. 1483-1537 | 0.1251 |
| **90** | RMSN | 1599-1635 vs. 1641-1693 | 0.1248 |
| **91** | Kurtosis | 1543-1585 | 0.1228 |
| **92** | Centroid | 1176-1242 vs. 1543-1585 | 0.1221 |
| **93** | Centroid | 1543-1585 vs. 1176-1242 | 0.1221 |
| **94** | Peak Frequency | 1020-1115 vs. 1543-1585 | 0.1213 |
| **95** | Peak Frequency | 1020-1115 | 0.1201 |
| **96** | Kurtosis | 1176-1242 vs. 1543-1585 | 0.1173 |
| **97** | Peak Frequency | 1020-1115 vs. 1599-1635 | 0.1122 |
| **98** | Kurtosis | 1641-1693 vs. 1176-1242 | 0.1115 |
| **99** | Peak Amplitude | 1483-1537 vs. 1176-1242 | 0.1090 |
| **100** | Peak Frequency | 1599-1635 vs. 1483-1537 | 0.1073 |
| **101** | Kurtosis | 1543-1585 vs. 1483-1537 | 0.1072 |
| **102** | RMSN | 1641-1693 vs. 1599-1635 | 0.1061 |
| **103** | Kurtosis | 1176-1242 vs. 1641-1693 | 0.1057 |
| **104** | Kurtosis | 1641-1693 vs. 1543-1585 | 0.1033 |
| **105** | Kurtosis | 1641-1693 | 0.1024 |
| **106** | Kurtosis | 1641-1693 vs. 1483-1537 | 0.0978 |
| **107** | Kurtosis | 1176-1242 | 0.0969 |
| **108** | Peak Frequency | 1483-1537 vs. 1020-1115 | 0.0961 |
| **109** | Peak Frequency | 1543-1585 vs. 1020-1115 | 0.09574 |
| **110** | Peak Frequency | 1599-1635 vs. 1020-1115 | 0.0950 |
| **111** | Peak Frequency | 1176-1242 vs. 1020-1115 | 0.0946 |
| **112** | Peak Frequency | 1483-1537 vs. 1599-1635 | 0.0944 |
| **113** | Kurtosis | 1483-1537 vs. 1641-1693 | 0.0927 |
| **114** | Peak Frequency | 1641-1693 vs. 1020-1115 | 0.0883 |
| **115** | RMSN | 1543-1585 | 0.0878 |
| **116** | Kurtosis | 1020-1115 vs. 1543-1585 | 0.0862 |
| **117** | Skew | 1599-1635 vs. 1176-1242 | 0.0851 |
| **118** | Peak Amplitude | 1483-1537 | 0.0805 |
| **119** | Peak Amplitude | 1483-1537 vs. 1641-1693 | 0.0789 |
| **120** | RMSN | 1599-1635 | 0.0755 |
| **121** | RMSN | 1641-1693 | 0.0754 |
| **122** | Kurtosis | 1020-1115 vs. 1483-1537 | 0.0712 |
| **123** | Peak Amplitude | 1641-1693 vs. 1483-1537 | 0.0695 |
| **124** | Kurtosis | 1020-1115 vs. 1599-1635 | 0.0676 |
| **125** | Kurtosis | 1543-1585 vs. 1599-1635 | 0.0662 |
| **126** | Kurtosis | 1599-1635 vs. 1543-1585 | 0.0647 |
| **127** | Kurtosis | 1020-1115 vs. 1641-1693 | 0.0637 |
| **128** | Skew | 1599-1635 | 0.0632 |
| **129** | Kurtosis | 1543-1585 vs.1599-1635 | 0.0631 |
| **130** | Centroid | 1641-1693 | 0.0615 |


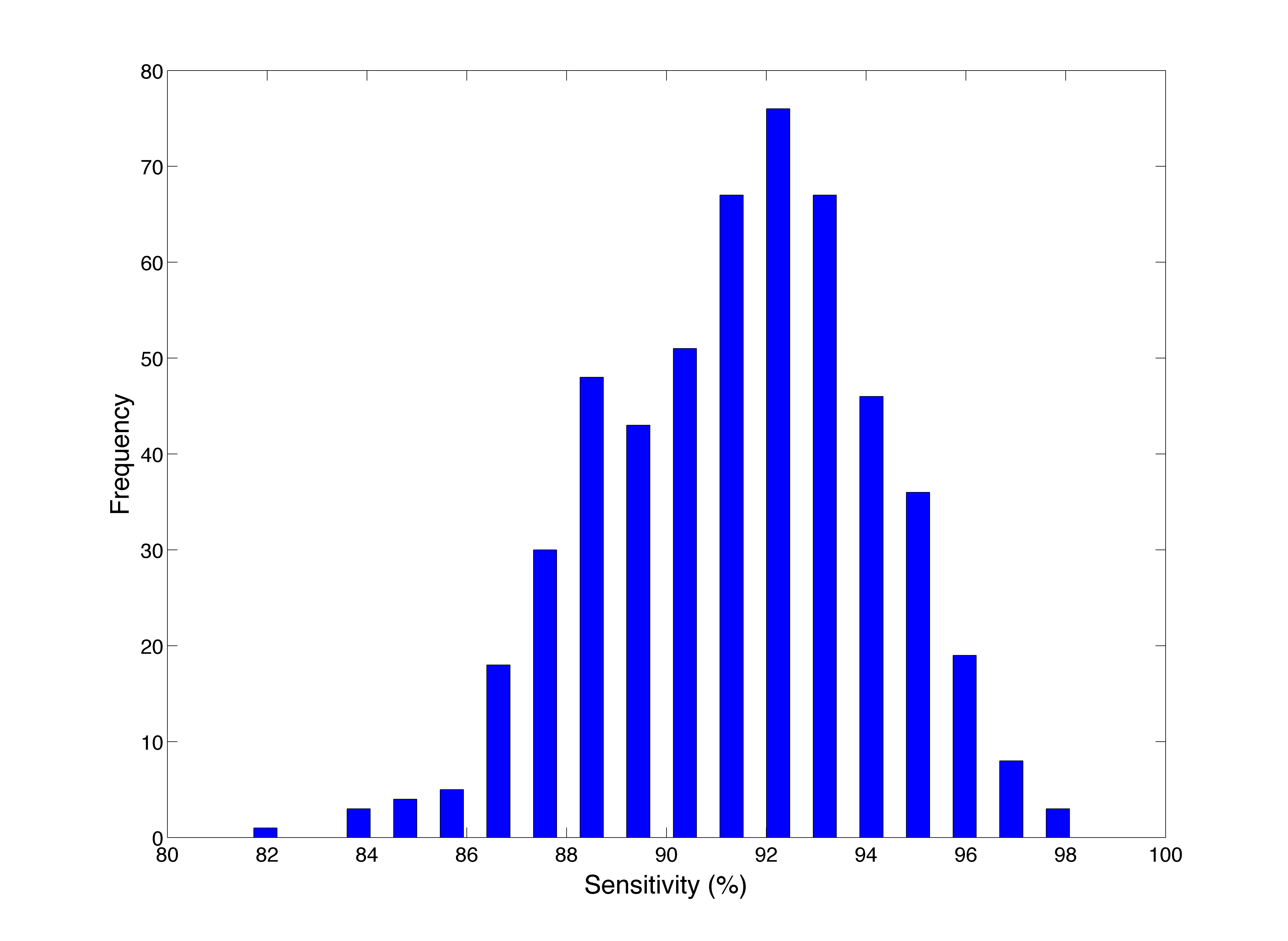

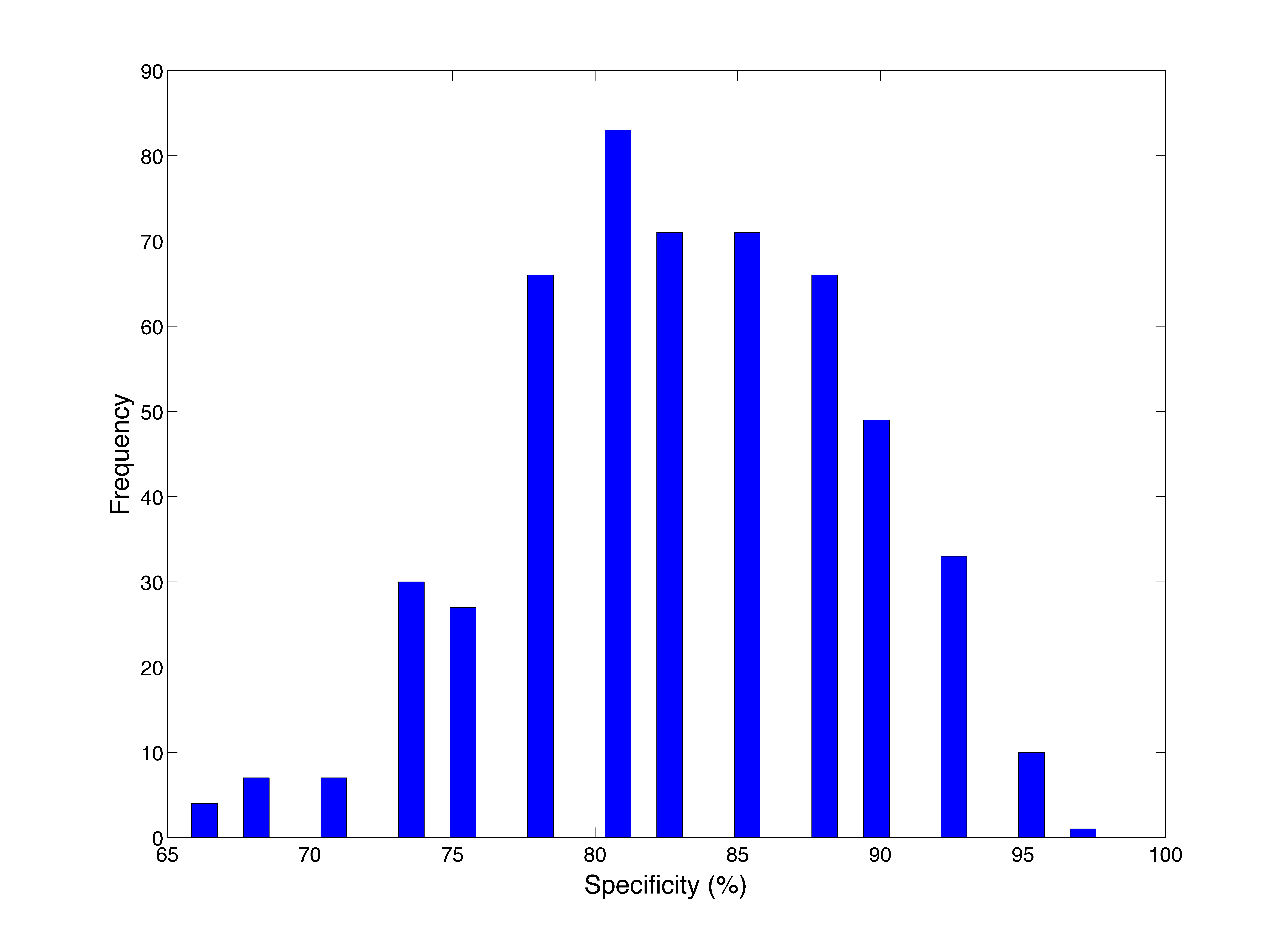

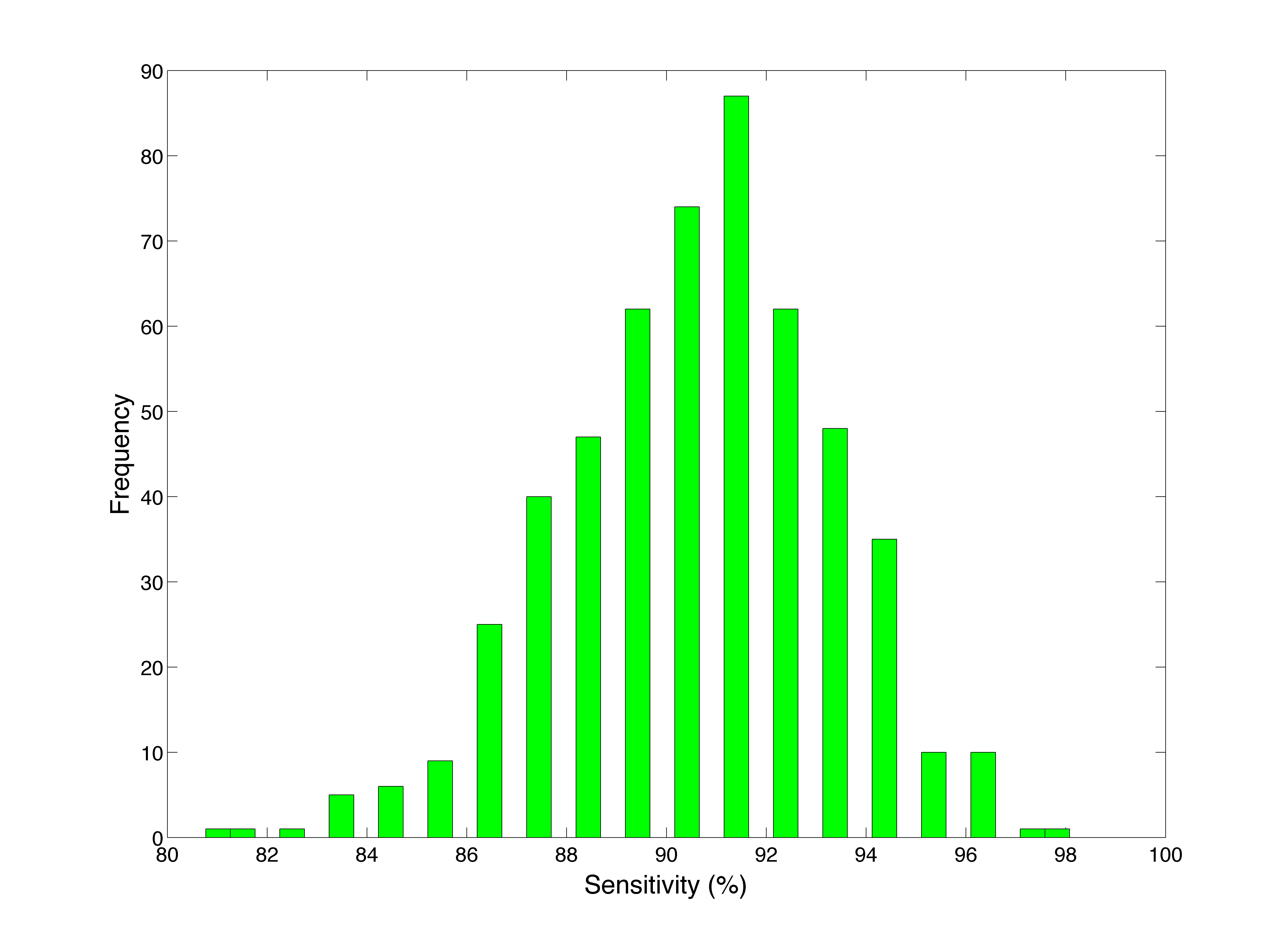

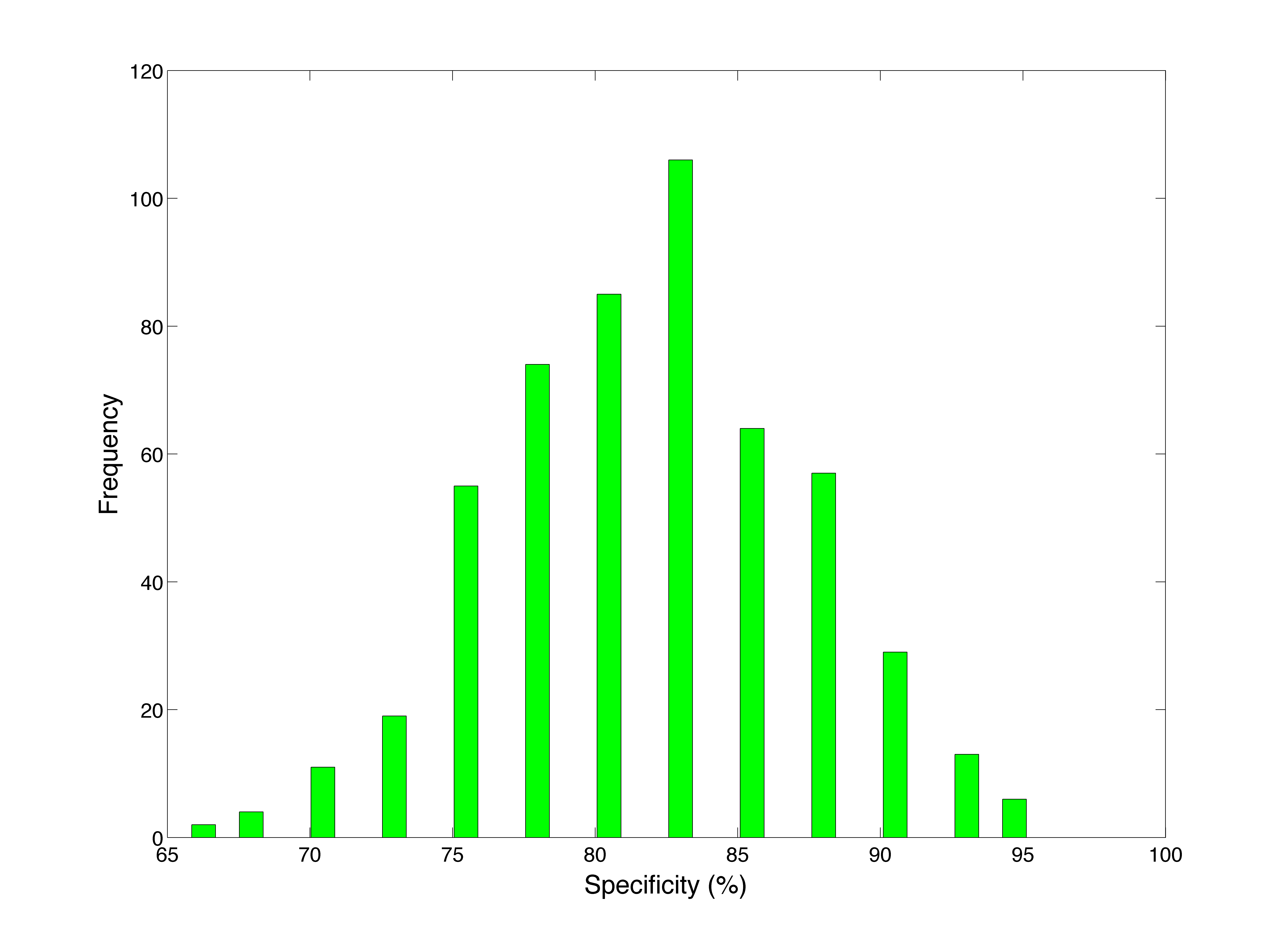

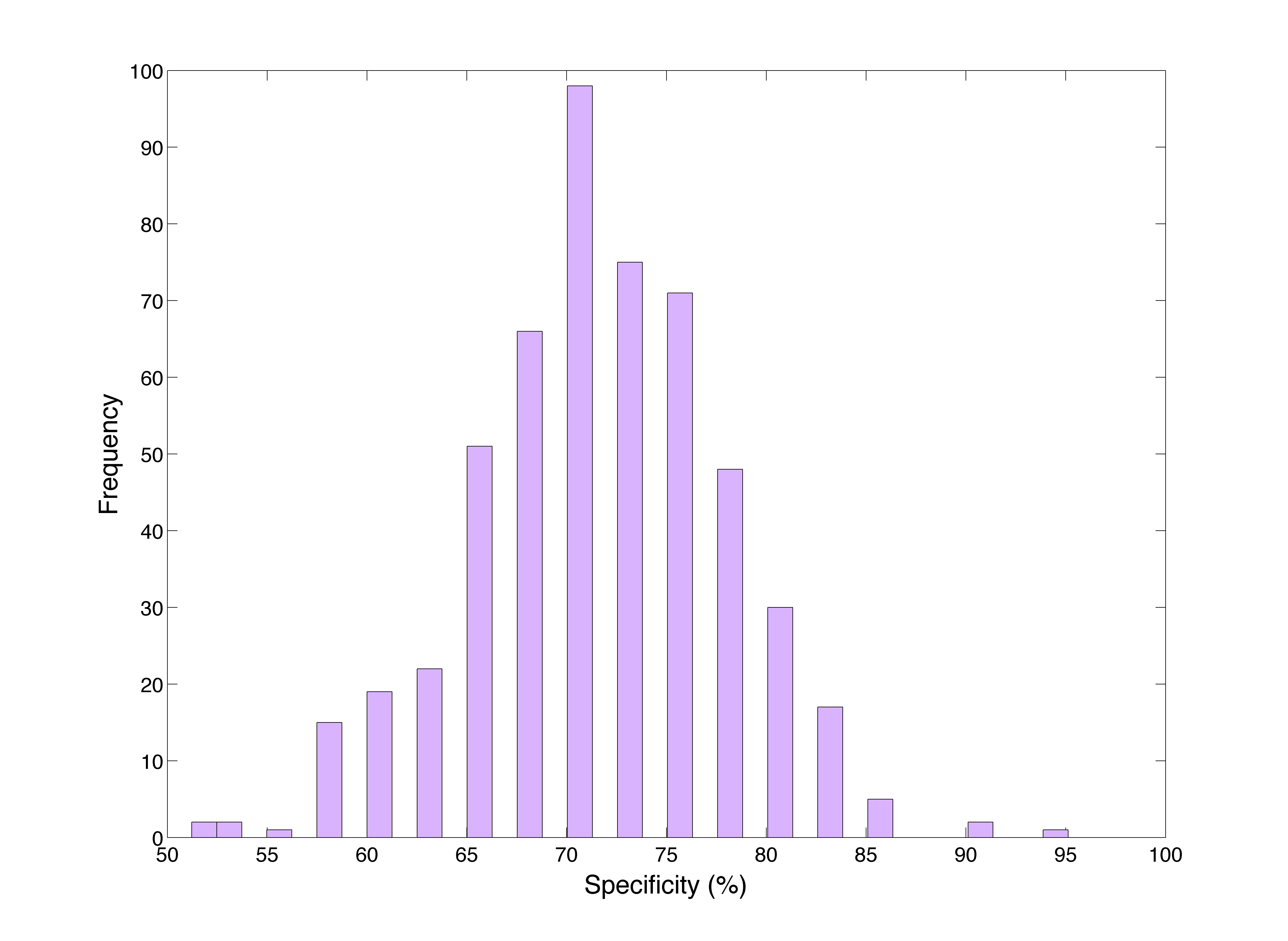

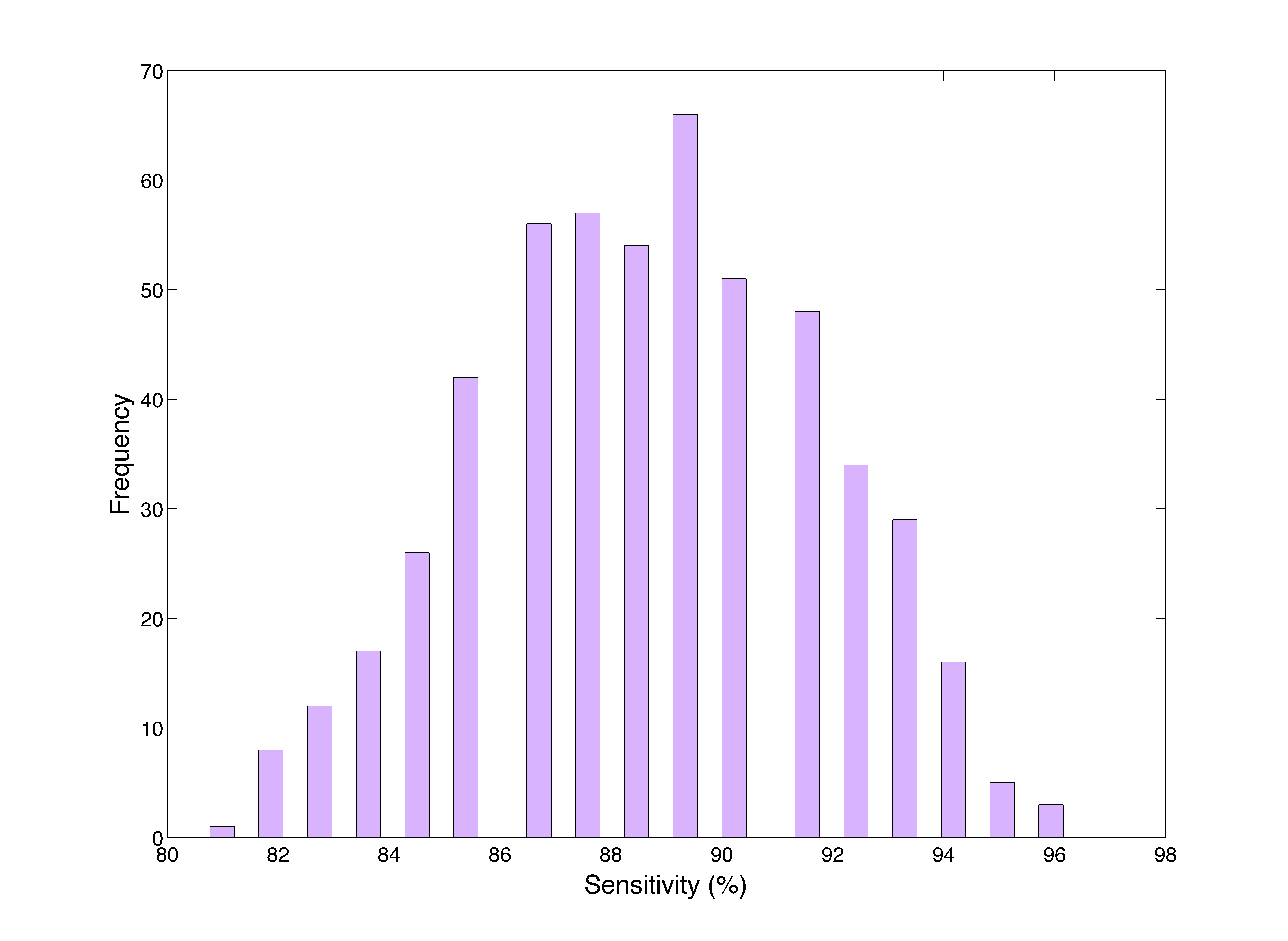


A

C

B

**S9.** Histograms showing the sensitivity and specificity results for 525 iterations of SVM conducted using (A) 130 features, (B) top 30 features and (C) top 2 features
